# Activation of NR1H3 attenuates the severity of septic myocardial injury by inhibiting NLRP3 inflammasome

**DOI:** 10.1002/btm2.10517

**Published:** 2023-04-06

**Authors:** Chao Deng, Qiong Liu, Huadong Zhao, Lu Qian, Wangrui Lei, Wenwen Yang, Zhenxing Liang, Ye Tian, Shaofei Zhang, Changyu Wang, Ying Chen, Yang Yang

**Affiliations:** ^1^ Department of Cardiovascular Surgery The First Affiliated Hospital of Xi'an Jiaotong University 277 Yanta West Road Xi'an 710061 China; ^2^ Key Laboratory of Resource Biology and Biotechnology in Western China, Ministry of Education, Faculty of Life Sciences and Medicine Northwest University 229 Taibai North Road Xi'an 710069 China; ^3^ Xi'an Key Laboratory of Cardiovascular and Cerebrovascular Diseases Xi'an No. 3 Hospital, The Affiliated Hospital of Northwest University, Faculty of Life Sciences and Medicine, Northwest University 10 Fengcheng Three Road Xi'an 710021 China; ^4^ Department of General Surgery Tangdu Hospital, The Airforce Medical University 1 Xinsi Road Xi'an 710038 China; ^5^ Department of Cardiothoracic Surgery The First Affiliated Hospital of Zhengzhou University 1 Jianshe East Zhengzhou 450052 China; ^6^ Department of Hematology The First Affiliated Hospital of Xi'an Jiaotong University 277 Yanta West Road Xi'an 710061 China

**Keywords:** Heart failure, Inflammation, NLRP3 inflammasome, NR1H3, Oxidative stress, Sepsis

## Abstract

Most sepsis deaths are due to the development of multiple organ failure, in which heart failure is a recognized manifestation of sepsis. To date, the role of liver X receptors α (NR1H3) in sepsis is still uncertain. Here, we hypothesized that NR1H3 mediates multiple essential sepsis‐related signalings to attenuate septic heart failure. Adult male C57BL/6 or Balbc mice and HL‐1 myocardial cell line were performed for in vivo and in vitro experiments, respectively. NR1H3 knockout mice or NR1H3 agonist T0901317 was applied to evaluate the impact of NR1H3 on septic heart failure. We found decreased myocardial expression levels of NR1H3‐related molecules while increased NLRP3 level in septic mice. NR1H3 knockout worsensed cardiac dysfunction and injury in mice subjected to cecal ligation and puncture (CLP), in association with exacerbated NLRP3‐mediated inflammation, oxidative stress, mitochondrial dysfunction, endoplasmic reticulum stress, and apoptosis‐related markers. The administration of T0901317 reduced systemic infection and improve cardiac dysfunction in septic mice. Moreover, Co‐IP assays, luciferase reporter assays, and chromatin immunoprecipitation analysis, confirmed that NR1H3 directly repressed NLRP3 activity. Finally, RNA‐seq detection further clarified an overview of the roles of NR1H3 in sepsis. In general, our findings indicate that NR1H3 had a significant protective effect against sepsis and sepsis‐induced heart failure.

## 1 INTRODUCTION

Sepsis leads to multiple organ dysfunction with unacceptably high mortality.^1^, ^2^ Sepsis‐induced cardiac dysfunction significantly increases the risk of cardiovascular events such as myocardial infarction.^3^ Previous studies on sepsis‐induced heart failure focused on the early abnormally amplified inflammatory factors, such as interleukin (IL)‐1β, IL‐6, and toll‐like receptor 4.^4^, ^5^ Though these studies indicated various inflammatory factors that play essential roles in sepsis, attempts to block a single inflammatory mediator have limited impact on mortality.^6^, ^7^ Research has also confirmed that in addition to excessive cardiac inflammation, the underlying mechanisms associated with sepsis and septic heart failure include oxidative stress, mitochondrial dysfunction, endoplasmic reticulum stress (ERS), and cardiomyocyte apoptosis. Unfortunately, despite decades of research, the pathogenesis of sepsis still remains limitation. It is pertinent to identify successful therapeutic drug that has clear impact on patient outcome.

Liver X receptors (LXRs), including NR1H3 (or LXRα) and NR1H2 (or LXRβ), belong to the ligand‐activated transcription superfamily. NR1H3 and NR1H2 share more than 78% sequence homology.^8^ Against these properties, activation of NR1H3 protect against myocardial ischemia reperfusion,^9^ cardiac hypertrophy,^10^ and diabetic cardiomyopathy.^11^ NR1H3 has also been unveiled to assume an organ‐protective role against liver injury in the cecal ligation and puncture (CLP) model as well as lipopolysaccharide (LPS) model.^12^, ^13^ These organ‐protective roles, including cardioprotective effects, may be primarily contributed to NR1H3's capability to attenuate inflammation.^14^

The NLRP3 inflammasome is one of the most research hotspots in the huge battery of molecules that regulate inflammation and immunity. NLRP3 serves as a platform to activate proinflammatory cytokine maturation and secretion. NLRP3 inflammasome assembly and activation can self‐cleavage to produce an active form of Caspase‐1 (pro‐Caspase‐1), promoting the release of proinflammatory cytokines including IL‐1β, IL‐18, and the alarmin high mobility group protein B1 (HMGB1), en route to inflammation of sepsis. Recently, Wang et al. revealed a critical regulatory role of NR1H3 in the metastasis of renal cell cancer via NLRP3 inflammasome.^15^ However, whether NR1H3 exerts a protective effect on septic heart failure via NLRP3 inflammasome remains unclear.

The primary goal of this study aimed to investigate whether NR1H3 confers cardioprotective effects against sepsis. First, we explored the expression level and function of NR1H3 and NLRP3 in CLP mice. Next, we investigated the mechanism of NR1H3 regulating the development of septic heart failure. Moreover, the involvement of NLRP3 in NR1H3‐regulated septic myocardial injury responses, if any, was determined. Through RNA‐seq and experiments verification, we identified transcriptome regulated by NR1H3 in septic mice. Taken together, our data suggest NR1H3 as a drug target for the prevention and management of septic heart failure.

## 2 RESULTS

### 2.1 CLP injury reduced myocardial expression levels of NR1H3‐related molecules

**FIGURE 1:**
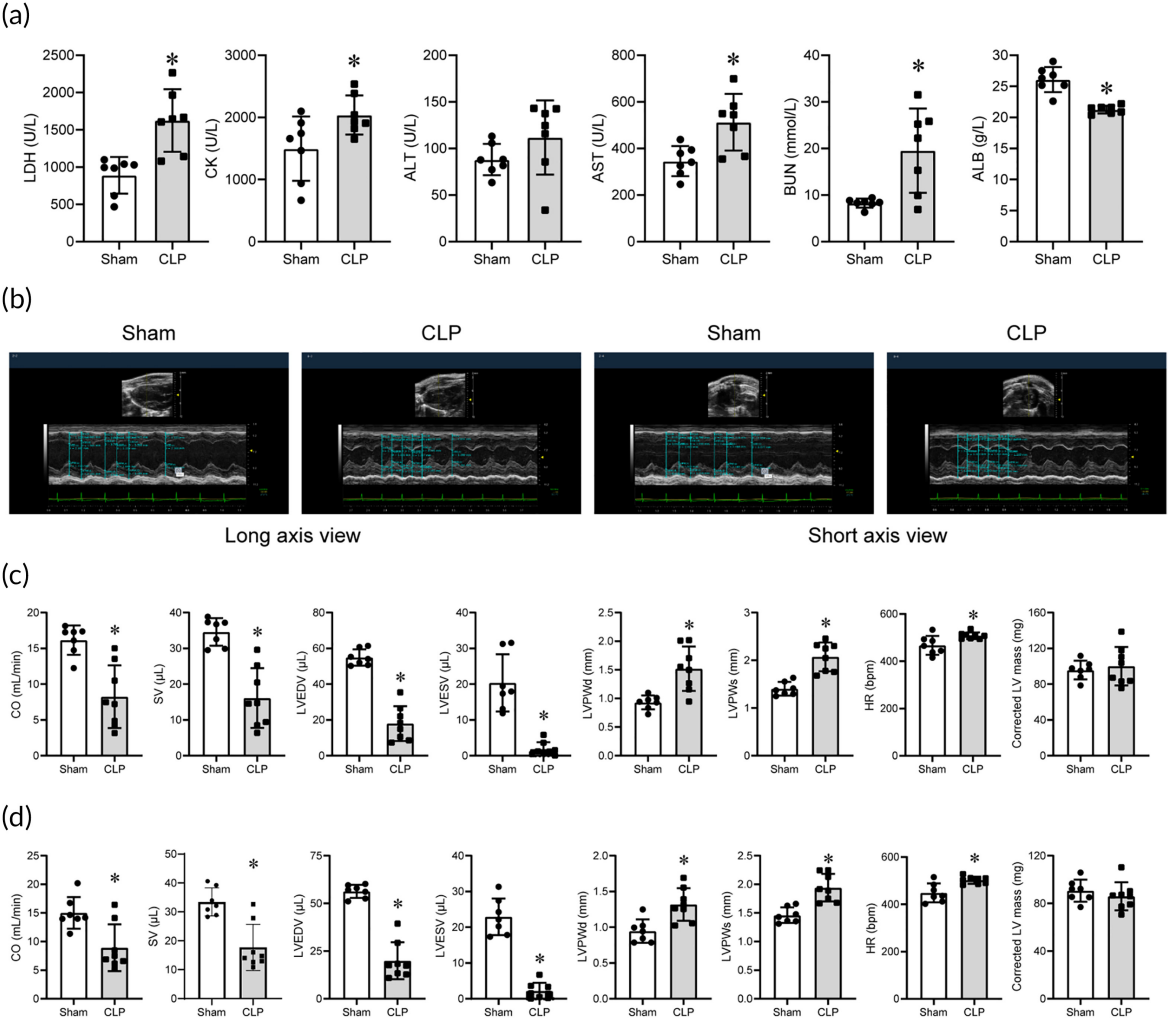
CLP injury led to systemic infection and myocardial dysfunction in mice. (a) Blood biochemistry parameters (*n* = 7 for each group). (b) Representative echocardiography images in the long‐axis and short‐axis view. (c) CO, SV, LVEDV, LVESV, LVPWd, LVPWs, HR, and corrected LV mass results in the long‐axis view (*n* = 7 for Sham, *n* = 8 for CLP). (d) CO, SV, LVEDV, LVESV, LVPWd, LVPWs, HR, and corrected LV mass results in the short‐axis view (*n* = 7 for Sham, *n* = 8 for CLP). Data are expressed as the mean ± SD. *p < 0.05 versus the Sham group. CLP, cecal ligation and puncture; CO, cardiac output; HR, heart rate; LVEDV, left ventricular diastolic volume; LVESV, left ventricular systolic volume; LVPWd, left ventricular end‐diastolic posterior wall thickness; LVPWs, left ventricular end‐systolic posterior wall thickness; SV, stroke volume.

First, we established CLP model, which mainly consists of two steps: cecal ligation and perforation (Figure S1A). As shown in Figure S1B,C, sepsis score increased while the anal temperature decreased significantly in CLP mice, proving the successful construction of septic model. The plasma concentrations of biochemical organ injury markers, including lactic dehydrogenase (LDH), creatine kinase (CK), aspartate aminotransferase (AST), and urea nitrogen (BUN) were increased in CLP mice (Figure 1a). Evaluation of cardiac function using echocardiography also displayed severe cardiac dysfunction in CLP mice, manifested as decreased cardiac output (CO), stroke volume (SV), left ventricular diastolic volume (LVEDV), left ventricular systolic volume (LVESV) while increased left ventricular end‐diastolic posterior wall thickness (LVPWd), left ventricular end‐systolic posterior wall thickness (LVPWs), and heart rate (HR) (Figure 1b–d). These data hinted the successful establishment of septic myocardial injury model.

**FIGURE 2:**
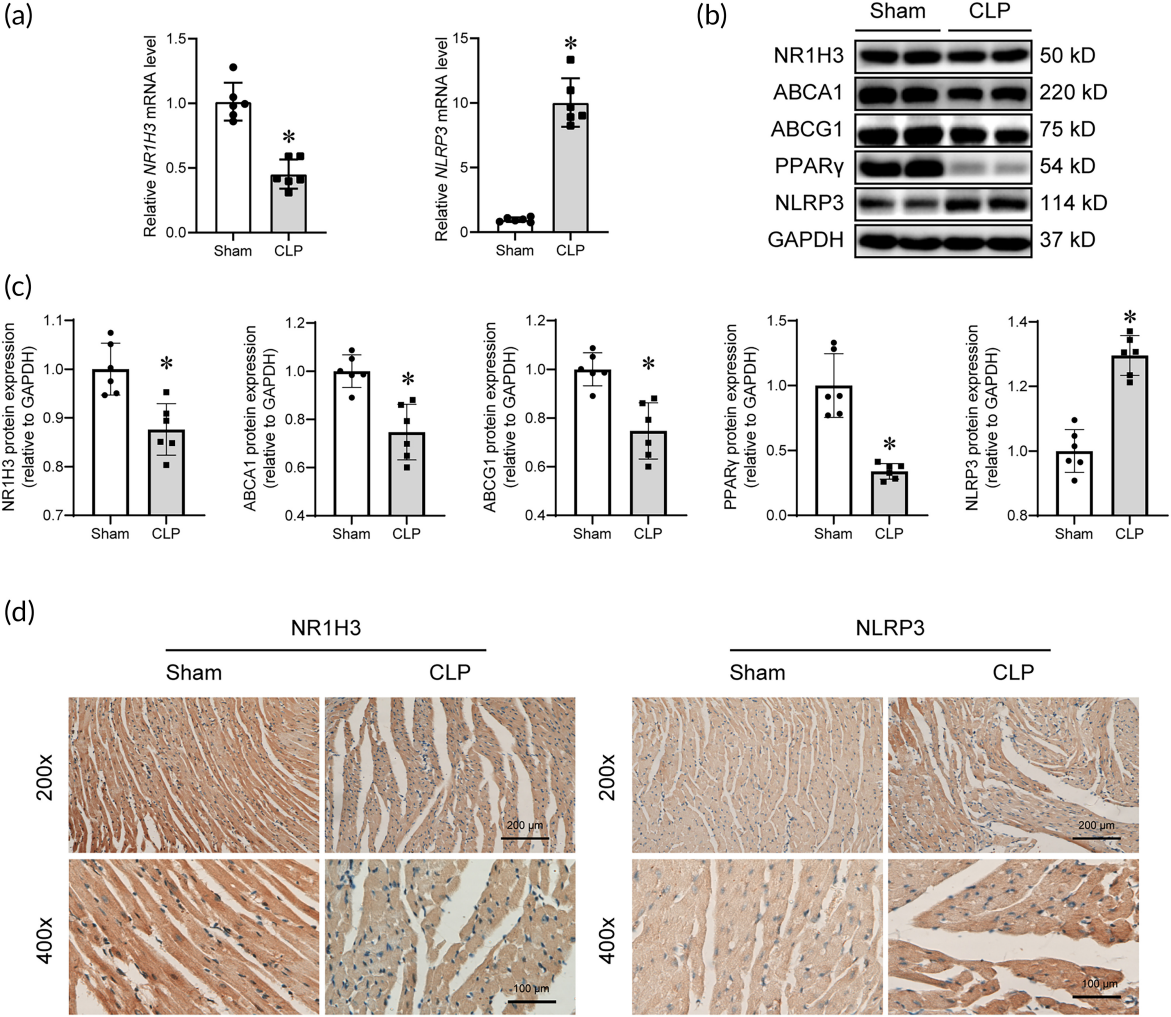
CLP injury reduced myocardial levels of NR1H3, ABCA1, ABCG1, and PPARγ while elevated NLRP3 level. (a) qPCR analysis of myocardial mRNA levels of NR1H3 and NLRP3. (b) Representative Western blot images of NR1H3, ABCA1, ABCG1, PPARγ, and NLRP3. (c) Quantitative analysis of these proteins in mouse heart tissues determined with GAPDH for normalization. (d) Representative photographs of IHC staining of NR1H3 and NLRP3. Data are expressed as the mean ± SD. *p < 0.05 versus the Sham group. *n* = 6 for each group. CLP, cecal ligation and puncture; qPCR, quantitative polymerase chain reaction.

Then, we found that CLP resulted in decreased mRNA and protein levels of myocardial NR1H3 while a significant increased myocardial NLRP3 (Figure 2a–c). IHC results of NR1H3 and NLRP3 indicated similar changes (Figure 2d). NR1H3 is a crucial regulator of the transcriptional control of cholesterol homeostasis, and our Western blot results revealed reduced myocardial levels of ABCA1, ABCG1, and PPARγ in CLP mice (Figure 2b,c). These data indicated the potential involvement of NR1H3 and NLRP3 in the pathological alterations of the septic heart.

### 2.2 The effects of NR1H3 knockout on myocardial inflammatory response, ERS, apoptosis, oxidative stress, and mitochondrial function signaling pathways in baseline mice

To elucidate the effects of NR1H3 on inflammatory response, ERS, apoptosis, oxidative stress, and mitochondrial function signaling pathways in the heart, NR1H3 knockout mice were constructed and confirmed by DNA and mRNA detection (Figure S2A,B). Next, the effects of NR1H3 knockout on myocardial inflammatory response, ERS, apoptosis, oxidative stress, and mitochondrial function‐related molecules were evaluated in the hearts of CLP‐free mice.

NLRP3 inflammasome is a critical factor in triggering the inflammatory response. As shown in Figure S3A,B, NR1H3 knockout enhanced the inflammatory response, as indicated by increased levels of not only NLRP3 and IL‐1β but also HMGB1 and IL‐6 in the myocardium of the mice. NR1H3 knockout also made a significant difference on ERS. The protein levels of ATF6, CHOP, and P‐PERK in the NR1H3 KO group were significantly increased, while those of GRP78 and PERK were decreased (Figure S3A,B). Additionally, enhanced apoptosis by significantly up‐regulating the pro‐apoptotic molecule Bax and down‐regulating the anti‐apoptotic Bcl‐2 was obserced in NR1H3 knockout mice (Figure S3A,B). Though NRF1 and TFAM protein levels were markedly increased in the NR1H3 KO group (Figure S3A,B), those of SIRT1, PGC‐1α, and UCP2 did not manifest notable discrepancy, suggesting that NR1H3 deletion might not have sufficient effect on mitochondrial function or oxidative stress‐related signaling pathways under the CLP‐free condition. These data indicated that NR1H3 knockout increased NLRP3‐related pathways and produced a negative effect on inflammatory response, ERS, and apoptosis‐related molecules even under undamaged conditions.

### 2.3 NR1H3 knockout reduced survival rate, elevated serum biomarker levels, and aggravated myocardial injury in septic mice

**FIGURE 3:**
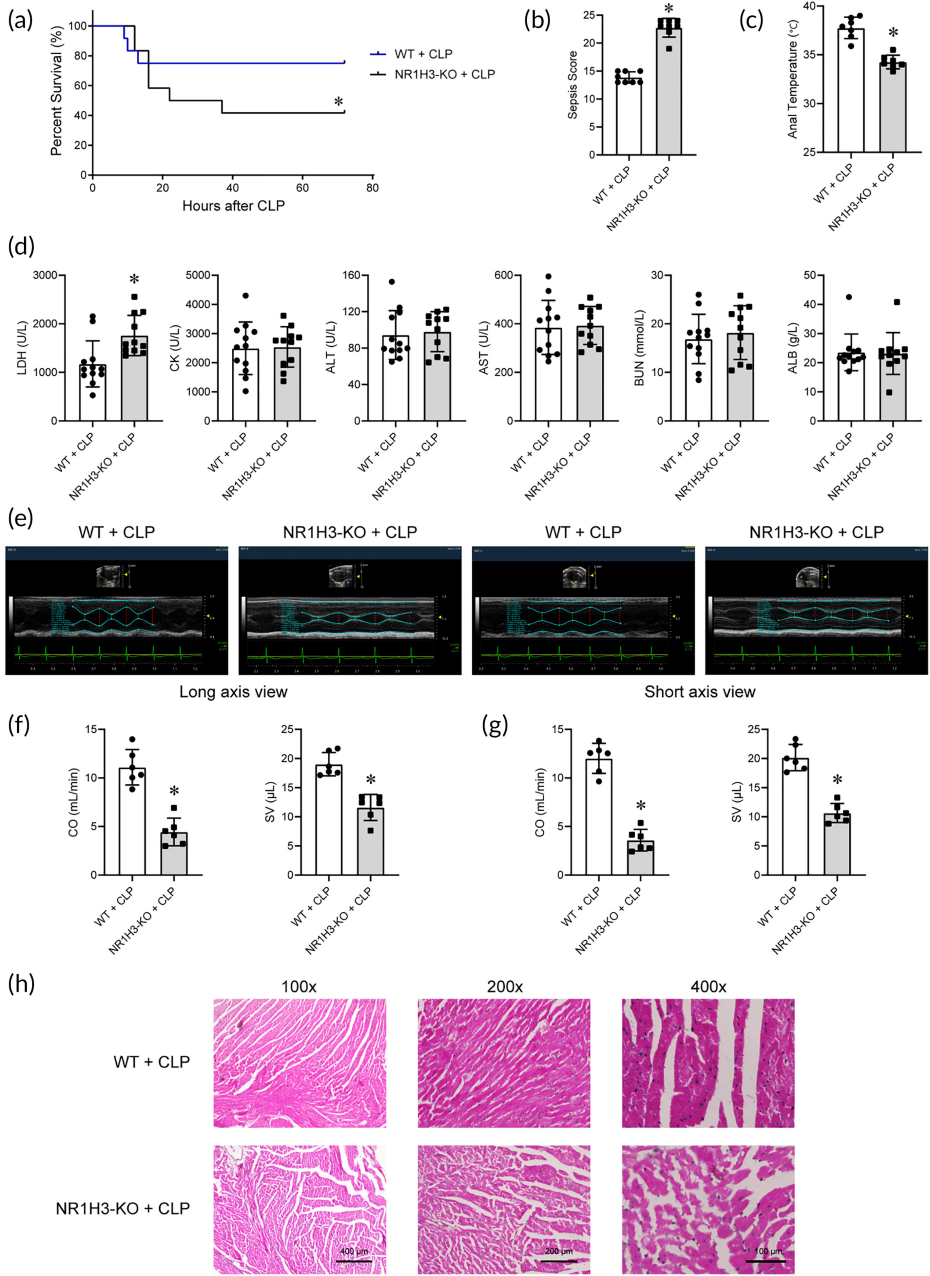
NR1H3 knockout reduced survival rate, elevated serum biomarker levels, and aggravated myocardial injury in septic mice. (a) KaplanMeier survival curves. Twelve animals for each group were used for comparison. Mortality was observed during 72 h of CLP. (b) The sepsis score (*n* = 8 for each group). (c) The anal temperature (*n* = 7 for each group). (d) Blood biochemical parameters (*n* = 12 for WT + CLP and *n* = 11 for NR1H3‐KO + CLP). (e) Representative echocardiography images of long axis and short axis. (f) CO and SV results in the long‐axis view (*n* = 6 for each group). (g) CO and SV results in the short‐axis view (*n* = 6 for each group). (h) Representative photographs of H&E staining of myocardial tissues. Data are expressed as the mean ± SD. *p < 0.05 versus the WT + CLP group. CLP, cecal ligation and puncture; CO, cardiac output; WT, wild type; SV, stroke volume.

To further clarify the role of NR1H3 in septic heart, the effects of NR1H3 deficiency on the survival, systemic infection, and cardiac dysfunction were examined in the heart of CLP mice. Aggravated CLP model was constructed to observe the survival rate (Figure S1D) during 72 h of CLP. Figure 3a showed approximately 25% of the wild type (WT) mice and 60% of the NR1H3 knockout mice died throughout 72 h of CLP (12 animals for each group were used for comparison), suggesting that NR1H3 deficiency significantly reduced the survival in CLP mice. This was also demonstrated by the increased sepsis score and reduced anal temperature in NR1H3 knockout mice subjected to CLP (Figure 3b,c). In particular, NR1H3 deficiency increased myocardial injury markers LDH levels (Figure 3d) while declined CO and SV in septic mice (Figure 3e–g). Additional echocardiographic result was shown in Figure S4A,B. H&E staining revealed severe myocardial tissue damage in the NR1H3‐KO + CLP group, as evidenced by disrupted myocardial fibers and destruction of cellular integrity (Figure 3h). Thus, NR1H3 knockout further exaggerated the septic myocardial injury.

### 2.4 The effects of NR1H3 knockout on NR1H3 pathway, inflammatory response, ERS, oxidative stress, apoptosis, and mitochondrial function‐related signaling pathways in septic mice

CLP injury induces an abnormal state, such as metabolic abnormalities and amplified inflammation, resulting in the changes of several essential signaling pathways related to sepsis.^16^ To clarify the mechanisms underlying the potent cardioprotective roles of NR1H3 against sepsis, the alterations of several essential pathways induced by sepsis were investigated.

**FIGURE 4:**
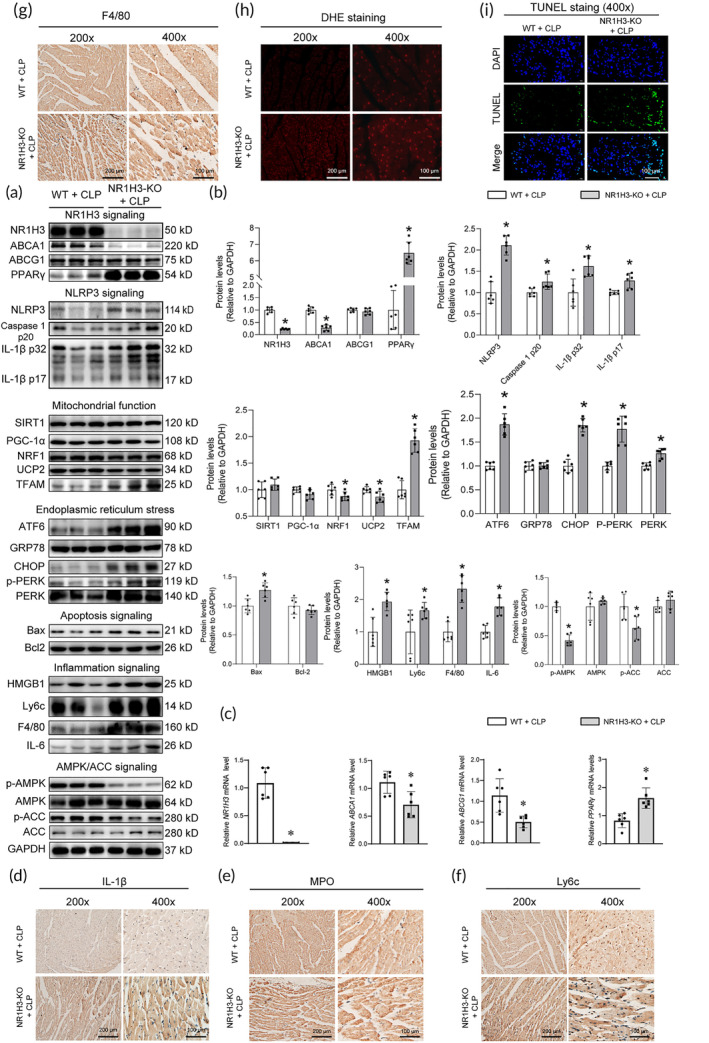
The effects of NR1H3 knockout on NR1H3 pathway, inflammation, ERS, oxidative stress, mitochondrial function, and AMPK pathway in septic mice. (a) The representative Western blot images of NR1H3, ABCA1, ABCG1, PPARγ, NLRP3, Caspase 1 p20, IL‐1β p32, IL‐1β p17, SIRT1, PGC‐1α, NRF1, UCP2, TFAM, ATF6, GRP78, CHOP, p‐PERK, PERK, Bax, Bcl2, HMGB1, Ly6c, F4/80, IL‐6, p‐AMPK, AMPK, p‐ACC, and ACC in mouse heart tissues. (b) Quantitative analysis of these proteins determined with GAPDH for normalization (*n* = 6 for each group). (c) qPCR analysis of myocardial mRNA levels of molecules involved in cholesterol metabolism including NR1H3, ABCA1, ABCG1, and PPARγ (*n* = 6 for each group). Representative photographs of IHC staining of (d) IL‐1β, (e) MPO, (f) Ly6c, and (g) F4/80. (h) Myocardial oxidative stress level was measured using a confocal microscope with in situ dihydroethidium stain. (i) Myocardial apoptosis was measured through TUNEL staining. Data are expressed as the mean ± SD. *p < 0.05 versus the WT + CLP group. CLP, cecal ligation and puncture; ERS, endoplasmic reticulum stress; qPCR, quantitative polymerase chain reaction; WT, wild type.

Significant changes in cholesterol levels during sepsis have been frequently reported.^17^ In this study, we found that the protein level of PPARγ was increased while NR1H3 and ABCA1 protein levels were decreased in the NR1H3‐KO + CLP group (Figure 4a,b). Consistently, NR1H3 knockout rose the PPARγ mRNA level while declined the mRNA levels of ABCA1 and ABCG1 of CLP mice (Figure 4c).

The inflammatory response is one of the major characteristics of sepsis.^18^ In this study, the protein levels of NLRP3, Caspase 1 p20, and IL‐1β in the myocardium of mice from the NR1H3‐KO + CLP group were significantly higher than those from the WT + CLP group, suggesting that NR1H3 deficiency could lead to activation of NLRP3 signaling pathways during sepsis (Figure 4a,b). In addition, the protein levels of inflammatory markers (Ly6c and F4/80) and proinflammatory factors (such as HMGB1 and IL‐6) were also elevated by NR1H3 knockout in septic mice (Figure 4a,b). IHC results further demonstrated this phenomenon, as indicated by overtly increased expressions of IL‐1β, MPO, Ly6c, and F4/80 in the NR1H3‐KO + CLP group (Figure 4d–g). These data together hinted that NR1H3 knockout aggravated myocardial inflammation in CLP mice.

Previous studies have also showed the key roles of ERS in septic injury.^19^ In this study, we found that NR1H3 knockout significantly elevated the expression of ATF6, CHOP, PERK, and p‐PERK in septic mice (Figure 4a,b). The data showed that NR1H3 knockout led to increased ERS in the myocardium of septic mice.

Together with inflammation and ERS, oxidative stress is involved in the pathophysiology of septic heart failure.^20^ As shown in Figure 4h, dihydroethidium (DHE) staining revealed much more ROS generation, as evidenced by the higher fluorescence intensity in the NR1H3‐KO + CLP group. Besides, the study also measured the effects of NR1H3 knockout on several essential signaling pathways, including apoptosis, mitochondrial function, and AMPK/ACC signaling pathway. Compared with the WT + CLP group, there were more TUNEL‐positive cells (green, Figure 4i) and increased pro‐aopotosis marker Bax (Figure 4a,b) in the NR1H3 knockout mice with CLP, indicating that the apoptosis of cardiomyocytes was increased in the NR1H3 knockout mice with CLP. NR1H3 knockout could also elevate the levels of TFAM while depressed the levels of NRF1, UCP2, p‐AMPK, and p‐ACC in septic mice (Figure 4a,b).

### 2.5 Administration of T0901317 reversed the elevation of sepsis score and serum biomarker and the deterioration of cardiac dysfunction in septic mice

**FIGURE 5:**
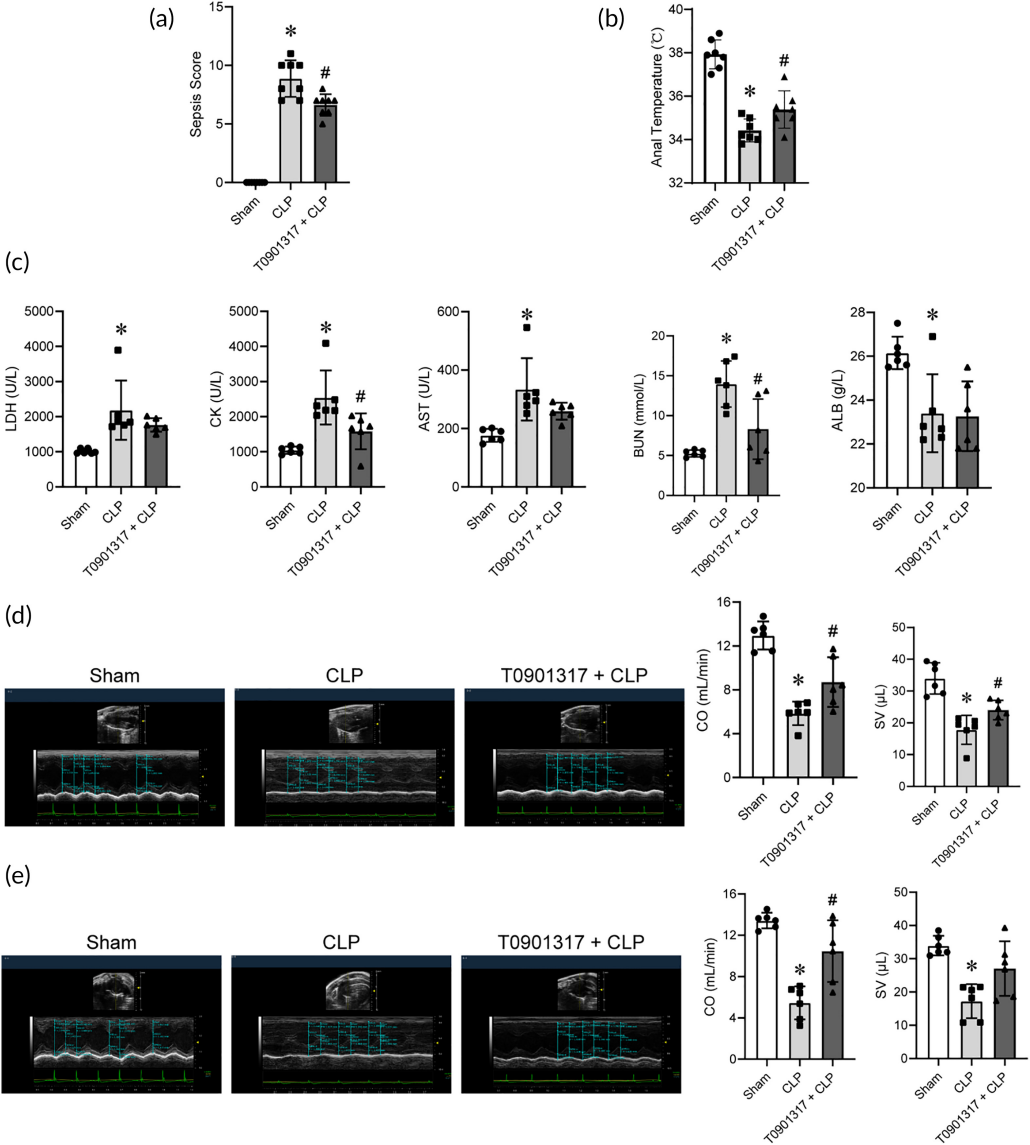
T0901317 treatment reversed the elevation of sepsis score and serum biomarker, and the worsening of cardiac dysfunction in septic mice. (a) The sepsis score (*n* = 8 for each group). (b) The anal temperature (*n* = 7 for each group). (c) Blood biochemical parameters (*n* = 6 for each group). (d) Representative echocardiography images and the CO and SV results in the long‐axis view (*n* = 6 for each group). (e) Representative echocardiography images and the CO and SV results in the short‐axis view (*n* = 6 for each group). Data are expressed as the mean ± SD. *p < 0.05 versus the Sham group, ^#^ *p* < 0.05 versus the CLP group. CLP, cecal ligation and puncture; CO, cardiac output; SV, stroke volume.

To further consolidate the findings, we investigated whether administration of the T0901317 (NR1H3 synthetic agonist) confered a cardioprotective effect in septic mice. As expected, T0901317 administration (10 mg/kg, i.p.) significantly decreased the sepsis score while increased the anal temperature of CLP mice (Figure 5a,b). The data also demonstrated that T0901317 administration could remarkably reduce the level of organ injury markers in CLP mice, including CK and BUN (Figure 5c). In particular, the CLP injury resulted in decreased CO and SV, while T0901317 produced a noticeable protective effects by restoring these cardiac function parameters (Figure 5d,e), indicating that T0901317 attenuated myocardial injury in CLP mice.

### 2.6 The effects of T0901317 treatment on several essential sepsis‐related signaling pathways in HL‐1 cardiomyocytes stimulated by LPS

**FIGURE 6:**
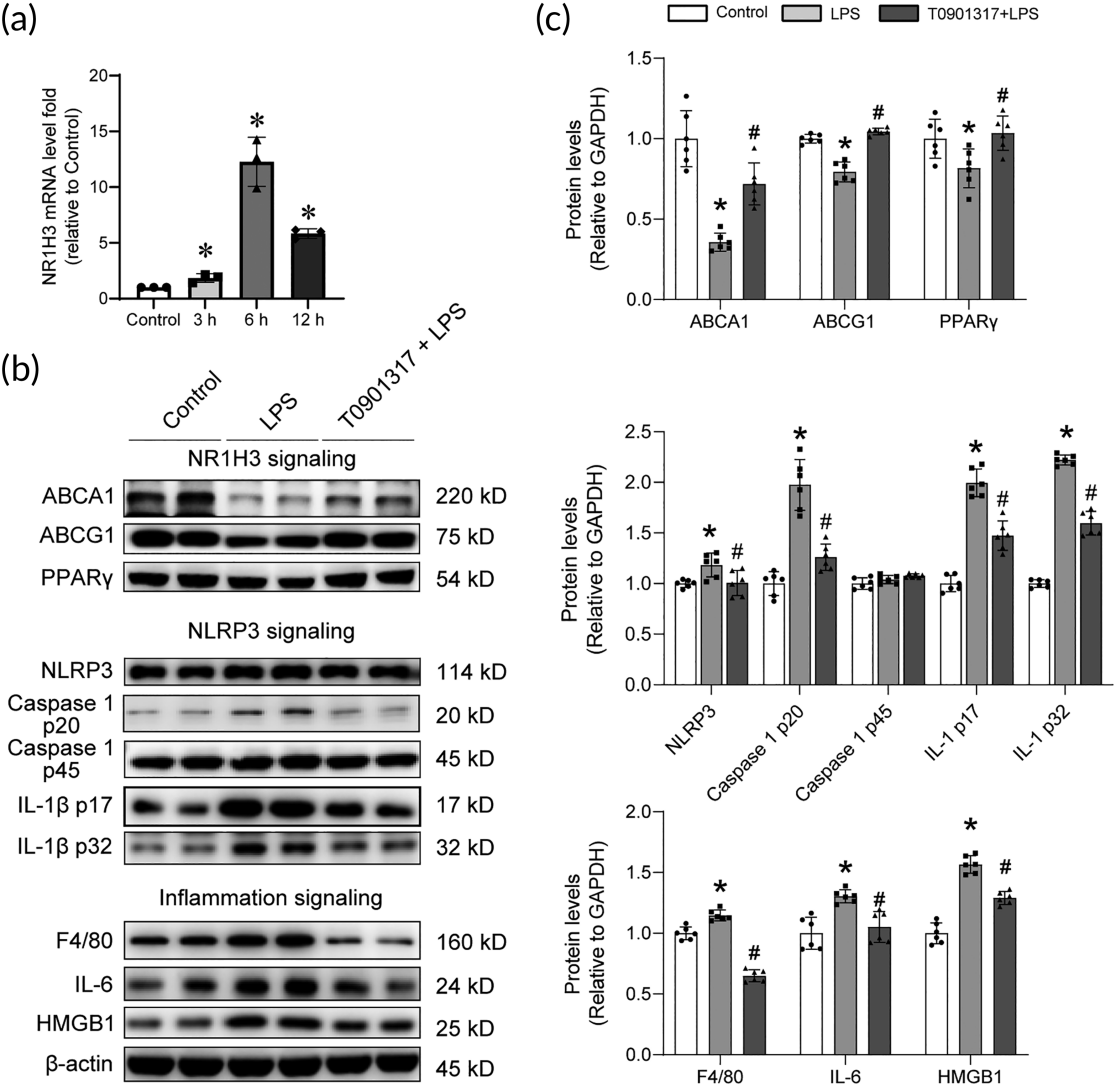
The effects of T0901317 treatment on several essential sepsis‐related signaling pathways in HL‐1 cells stimulated by LPS. (a) NR1H3 mRNA levels at different time points after T0901317 addition in HL‐1 cells (*n* = 3 for each group). (b) Representative Western blot images of ABCA1, ABCG1, PPARγ, NLRP3, Caspase 1 p20, Caspase 1 p45, IL‐1β p17, IL‐1β p32, F4/80, IL‐6, and HMGB1 in HL‐1 cells. (c) Quantitative analysis of these proteins in HL‐1 cells determined with β‐actin for normalization (*n* = 6 for each group). Data are expressed as the mean ± SD. *p < 0.05 versus the Control group, ^#^ *p* < 0.05 versus the LPS group. LPS, lipopolysaccharide.

To determine whether T0901317 yielded protective phenotypes on the LPS‐stimulated cell, first, the effects of 10 μM T0901317 on the expression and activation of NLRP3 pathways at different time points after T0901317 addition was evaluated. As shown in Figure 6a, the mRNA level of NR1H3 increased significantly at 6 h in HL‐1 cells after T0901317 treatment. Therefore, 6 h was chosed as the time point to evaluate the effects of T0901317 addition in the following experiments. We also found that T0901317 reversed the abnormal cholesterol metabolism, NLRP3 pwathway, and inflammatory response, as shown by not only increases in ABCA1, ABCG1, and PPARγ, but also decreases in NLRP3, Caspase 1 p20, IL‐1β, F4/80, IL‐6, and HMGB1 in HL‐1 cells after LPS challenge (Figure 6b,c). The above results hinted that the activation of NR1H3 might provide potential positive effects against LPS injury by regulating cholesterol metabolism and inflammation‐related signaling pathways.

### 2.7 Nigerin could reverse the protective role of T0901317 in HL‐1 cells stimulated by LPS

In order to further explore whether gain‐of‐function of NLRP3 will affect NR1H3's effects, HL‐1 cells were treated with nigerin (NLRP3 agonist) and T0901317. As shown in Figure S5A, no changes in cell viability were observed after different concentrations of nigerin treatment (2, 4, 6, 8, 10 μM). However, the mRNA levelof NLRP3 reached the highest levels at 6 h in HL‐1 cells after 10 μM nigerin treatment (Figure S5B). Then, HL‐1 cells were exposed to 10 μM nigerin and 10 μM T0901317 for 6 h prior to exposure to LPS injury in order to observe the effect of nigerin on the protective role of T0901317 in HL‐1 cells. Nigerin abolished the T0901317‐induced rise in NR1H3 level and decline in NLRP3 level (Figure S5C). Meanwhile, nigerin also reversed the T0901317‐induced decrease of proinflammatory factor IL‐6 (Figure S5C). These results indicated that NLRP3 activation might reverse the protective role of T0901317 in HL‐1 cells stimulated by LPS, at least partly.

### 2.8 NR1H3 bound to NLRP3 and repressed NLRP3 transcription

**FIGURE 7:**
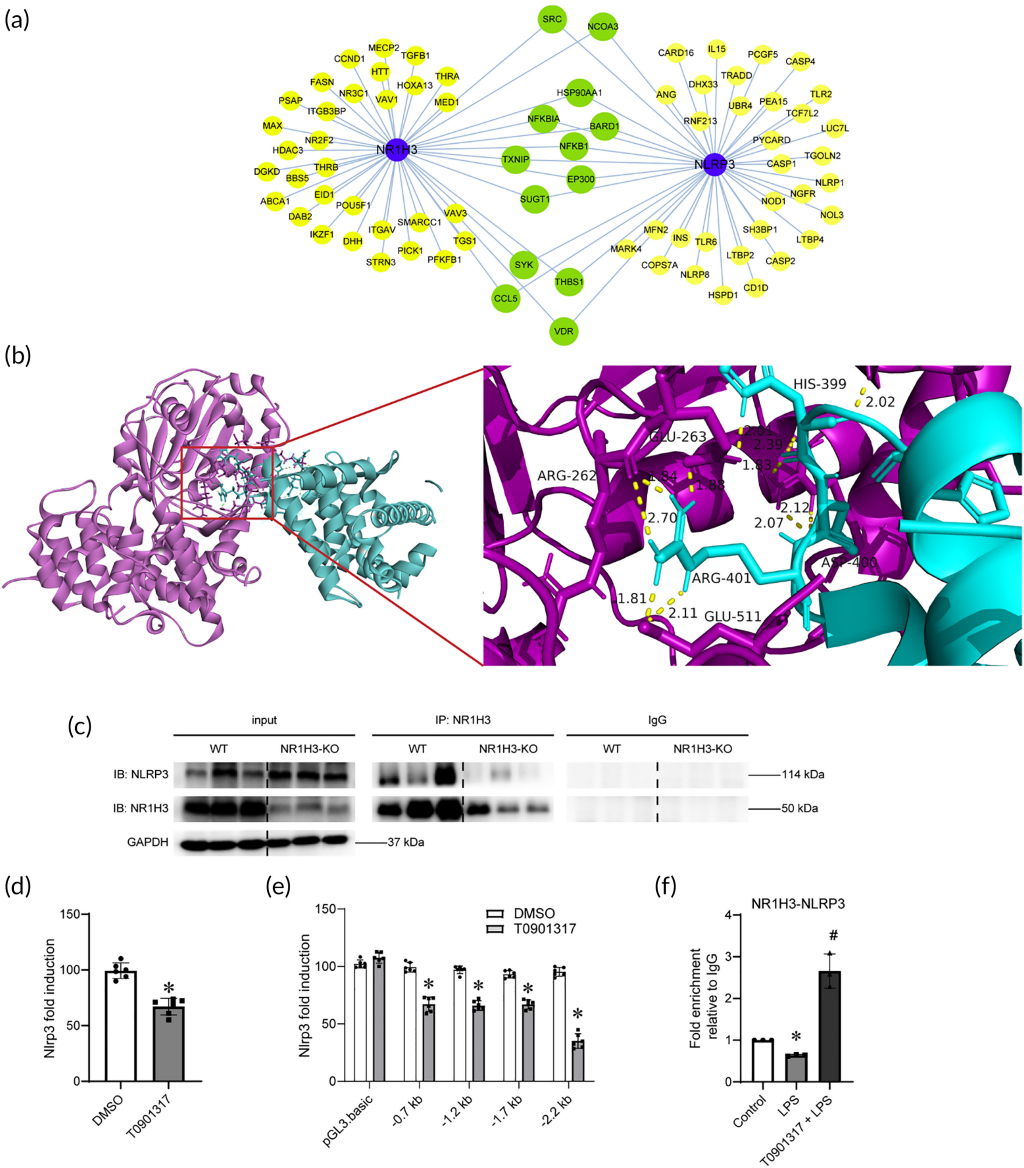
NR1H3 bound to NLRP3 and repressed NLRP3 transcription. (a) Integrated Interactions Database analysis of the relationship among NR1H3 and NLRP3. (b) The docking results of CLUSPro software. (c) Co‐IP results showing that NR1H3 has an interaction with NLRP3. (d) Luciferase reporter assays showing that NR1H3 activation downregulates NLRP3 transcription in HL‐1 cells. HL‐1 cells were transfected with the 2.2 kb NLRP3 reporter followed by treatment with T0901317 (or DMSO) for 6 h and then injured by LPS. (e) Luciferase reporter assays with distinct NLRP3 reporters in HL‐1 cells. HL‐1 cells were transfected with blank pGL3.basic and Nlrp3 reporter (i.e., 0.7, 1.2, 1.7, or 2.2 kb) followed by treatment with T0901317 (or DMSO) for 6 h and then injured by LPS. (f) ChIP assay showed recruitment of NR1H3 to NLRP3 promoter in HL‐1 cells. HL‐1 cells were pretreated with T0901317 or vehicle for 6 h, followed by LPS administration. The concentrations of T0901317 and LPS for cell treatment were 10 μM and 10 μg/mL, respectively. Data are mean ± SD. *p < 0.05 versus the DMSO or control group, ^#^ *p* < 0.05 versus the LPS group. *n* = 3. ChIP, chromatin immunoprecipitation; LPS, lipopolysaccharide.

NLRP3, the platform of proinflammatory cytokine maturation and secretion, plays a critical role in sepsis.^21^ As mentioned earlier, CLP induced a dramatical increase in NLRP3 level, while NR1H3 knockout further elevated the level under CLP condition. Then, a series of experiments were performed to explore whether NR1H3 has an interaction with NLRP3. Protein–protein interactions network diagram exhibited that NR1H3 may serve as a regulator of NLRP3 through various downstream targets, such as chemokines CCL5, spleen tyrosine kinase, and vitamin D receptor (Figure 7a). Through ZDOCK and Cluspro docking simulation experiments, we further verified the interaction between NLRP3 and NR1H3. Cluspro results showed a complex formed by the two proteins NLRP3 and NR1H3 (Figure 7b). The ZDOCK results showed that the docking binding energy of NLRP3 and NR1H3 was −9.3 kcal/mol, and the ZDOCK score was 1000.523. To validate whether there has a direct relationship between the NR1H3 and NLRP3, Co‐IP assays were conducted. The results demonstrated that NLRP3 (IB) could be detected when NR1H3 acted as the target protein (IP) in the WT group, while NR1H3 knockout led to a decrease of NLRP3 level (Figure 7c), denoting a direct interaction between NR1H3 and NLRP3. In luciferase reporter assays with HL‐1 cardiomyocytes, NR1H3 was found to repress the transcription activity of NLRP3 (Figure 7d,e). Promoter analysis of NLRP3 revealed that a 500‐bp region (−1700 to −2200 bp) was responsible for the repressor activity of NR1H3. Additionally, the chromatin immunoprecipitation (ChIP) assays showed that the enrichment level of NR1H3 in the NLRP3 promoter was significantly increased in the T0901317 + LPS group, supporting the direct interaction between NR1H3 and NLRP3 promoter *in vitro* (Figure 7f). These results together confirmed the regulatory relationship between NR1H3 and NLRP3 in the myocardium.

### 2.9 RNA‐sequencing identified transcriptome regulated by NR1H3 in septic mice

**FIGURE 8:**
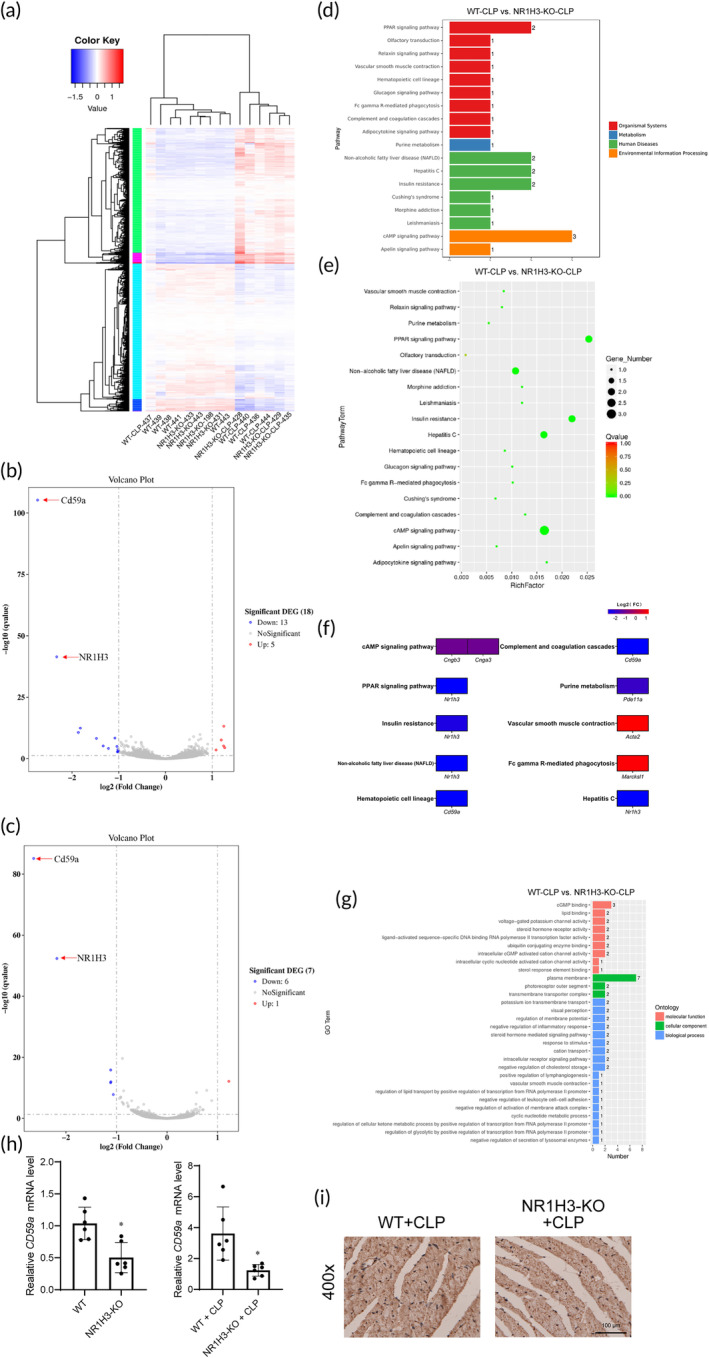
RNA‐seq identified transcriptome regulated by NR1H3 in septic mice. (a) Cluster analysis of differential genes in each group. (b) Differentially expressed mRNAs between WT and NR1H3‐KO mice were displayed by volcano plots. (c) Differentially expressed mRNAs between WT‐CLP and NR1H3‐KO + CLP mice were displayed by volcano plots. The blue and red parts indicated >2‐fold decreased and increased expression of the dysregulated mRNAs in cardiac tissues, respectively (*p* < 0.05). (d and e) KEGG pathways analysis of the WT‐CLP group and NR1H3‐KO‐CLP group. (f) Expression of differential genes based on pathways. The blue and red parts indicated >2‐fold decreased and increased expression of the dysregulated mRNAs in cardiac tissues, respectively (*p* < 0.05). (g) GO analysis of the WT‐CLP group and NR1H3‐KO‐CLP group. (h) qRT‐PCR analysis of CD59a by normalizing to β‐actin. (i) Representative photographs of IHC staining of CD59a. Data are expressed as the mean ± SD. *p < 0.05 versus the WT or WT + CLP group, *n* = 6 for each group. CLP, cecal ligation and puncture; GO, Gene Ontology; KEGG, Kyoto Encyclopedia of Genes and Genomes; qRT‐PCR, Quantitative real‐time polymerase chain reaction; WT, wild type.

To fully characterize the transcriptional changes and the downstream molecular events of NR1H3 deficiency that exacerbated heart failure in sepsis, we performed RNA‐sequencing experiments on hearts from septic mice of WT or NR1H3 KO. The RNA‐sequencing results suggested that the alteration of these genes is dependent on NR1H3 in pathological remodeling (Figure 8a). Among these differentially expressed genes, volcanic map showed that NR1H3 knockout significantly down‐regulated NR1H3 and CD59a levels in mouse myocardium (Figure 8b). Besides, we compared the RNA‐seq of NR1H3‐KO + CLP with WT + CLP and found the similar results (significantly down‐regulated CD59a and NR1H3 in the NR1H3‐KO + CLP group, Figure 8c). We then performed the Kyoto Encyclopedia of Genes and Genomes pathway analysis between the NR1H3‐KO + CLP group and WT + CLP group, which further revealed that NR1H3 might regulate pathways pertinent to cAMP signaling pathway, PPAR signaling pathway, vascular smooth muscle contraction, Fc gamma R‐mediated phagocytosis, etc. (Figure 8d‐f). The Gene Ontology analysis determined the functional changes after CLP. Differential expressed genes participated in multiple biological processes. Among them, biological molecular function includes cAMP binding, lipid binding, voltage‐gated potassium channel activity, etc.; the cellular component is involved in plasma membrane, photoreceptor outer segment, transmembrane transporter complex; the biological process is involved in potassium ion transmembrane transport, visual perception, regulation of membrane potential, etc. (Figure 8g). Furthermore, the represented gene were validated by quantitative polymerase chain reaction (qPCR) and IHC staining. CD59a is a glycosylphosphatidylinositol anchored membrane‐bound protein. CD59a is widely distributed in various organs and tissues of the human body, and has been proven to play a positive role in a variety of inflammatory diseases, such as pneumonia, nephritis, and allergic encephalomyelitis.^22^, ^23^ Consistent with our results of RNA‐seq, NR1H3 knockout markedly down‐regulated the mRNA levels of NR1H3 and CD59a either in the heart of baseline or CLP mice (Figure 8h). Similarly, the CD59a protein level was also declined in the NR1H3‐KO + CLP mice (Figure 8i). Taken together, these results provided an overview role of NR1H3 in response to sepsis.

## 3 DISCUSSION

Septic heart failure remains a main cause of death in intensive care units.^24^ Despite significant advances in understanding the pathophysiological of septic heart failure, the detailed molecular mechanisms have not been fully elucidated.^25^ NR1H3 is known as a nuclear receptor involved in regulating lipid and glucose metabolism and inflammation. Besides, activation of NR1H3 has been proved to attenuate pathological cardiac hypertrophy^26^ and ischemia/reperfusion injury,^9^ suggesting that NR1H3 may be identified as a critical cardiac transcriptional regulator. A surprising role of NR1H3 in sepsis has also been noted. Botez et al. demonstrated that NR1H3 plays a positive role in the pathogenesis of liver injury induced by CLP.^27^ Another study proved that NR1H3 was involved in the anti‐inflammatory role of Saikosaponin a on LPS‐induced sepsis.^28^ Based on the above background, this study provided new insights into the regulatory role of NR1H3 against septic heart failure. The results identified that NR1H3 could be a node in the complex regulatory network of septic heart failure. Specifically, NR1H3 deletion alone increased NLRP3‐related pathways and produced a significant negative effect on inflammatory response, ERS, and apoptosis‐related molecules. To further confirm the role of NR1H3 in septic heart failure, NR1H3 knockout mice were subjected to CLP surgery. NR1H3 knockout led to a reduced survival rate within 72 h CLP, accompanied by abnormal cardiac function in septic mice. The above data indicated that NR1H3 deletion could indeed aggravate sepsis and septic heart failure. To clarify the mechanisms underlying the potent cardioprotective role of NR1H3 against sepsis and septic heart failure, the alterations of several essential signaling pathways was then investigated.

One of the metabolic abnormalities that accompany septic myocardial injury is the alteration in cholesterol metabolism. Decreased levels of plasma cholesterol are associated with poor prognosis of patients with sepsis.^29^ Cholesterol metabolism is regulated by various genes, including PPARγ, NR1H3, ABCA1, and ABCG1. PPARγ, identified as a critical nuclear transcription factor of cholesterol,^30^ has been shown to increase ABCA1 or ABCG1 expression via the induction of NR1H3 expression in cholesterol‐loaded macrophages. Thus, the PPARγ‐NR1H3‐ABCA1/ABCG1 is a crucial pathway for cholesterol metabolism. PPARγ has also been confirmed to play an essential regulatory role in inflammation and sepsis.^31^, ^32^ Jiang et al. found that in H9c2 cells, the activation of PPARγ ameliorates doxorubicin‐induced inflammation and oxidative stress.^31^ Liu et al. proved that PPAR γoverexpression reduces the inflammatory response and inhibits apoptosis via inhibiting phosphatase and tensin homolog (PTEN)/β‐catenin pathway in sepsis‐induced acute lung injury.^32^ NR1H3‐mediated ABCA1/ABCG1‐dependent cholesterol efflux from macrophages is also involved in the occurrence and progression of cardiovascular diseases, such as atherosclerosis^33^ and cardiac hypertrophy.^34^ In this study, the results showed that NR1H3 knockout could inhibit ABCA1‐related cholesterol metabolism pathway and increase the level of PPARγ in septic mice. Since NR1H3 activity/expression is regulated by the PPARγ network as discussed above, and NR1H3 knockout mice behave very similar phenotype with WT mice according to our observation, we speculated that PPARγ upregulation may be compensatory responses to NR1H3 deficiency that ensure normal growth of the mice. Likewise, we found that the up‐regulated TFAM and NRF1 in NR1H3 knockout mice might also be the compensatory response to maintain the normal life activities of mice.

A subgroup of patients with sepsis have excessive inflammation, oxidation, and an exaggerated immune response, which leads to an inflammatory cytokine storm syndrome.^35^, ^36^ Therefore, it is promising to seek key inflammatory molecular targets during septic myocardial injury. In line, we first demonstrated that NR1H3 knockout significantly intensified the inflammatory response, as evidenced by increased levels of inflammatory markers (IL‐1β, MPO, Ly6c, and F4/80) and inflammatory cytokines (HMGB1 and IL‐1β). The RNA‐seq results further clarified the role of NR1H3 in regulating a transcriptional profile. A variety of signaling pathways were involved, such as the pathways pertinent to cAMP signaling pathway, PPAR signaling pathway, Fc gamma R‐mediated phagocytosis, CD59a‐induced inflammation, etc., which is consistent with its role in the inflammatory response during sepsis.

In order to clarify the precise mechanisms of NR1H3 against inflammation during sepsis, we specifically focused on the inflammation‐related NLRP3 signaling pathway. The NLRP3/Caspase‐1/IL‐1β axis has emerged as a critical signaling pathway of the progression of inflammation.^37^, ^38^ As stated by Lei et al., NR1H3 agonist T0901317 ameliorates amyloid β‐induced retinal inflammatory response by inhibiting the NF‐κB signaling and the production of NLRP3, Caspase‐1, and IL‐1β.^39^ Here, the study also established a critical role of the NLRP3 signaling pathway regulated by NR1H3 in septic myocardial injury. In the *in vivo* research, NR1H3 knockout can increase the expression of NLRP3, IL‐1β, and Caspase‐1 both in the baseline condition and in septic mice. However, NLRP3 agonist nigerin could reverse the protective role of T0901317 in HL‐1 cells stimulated by LPS. Furthermore, a series of assays, including the Co‐IP method, ChIP assays, and luciferase reporter assays, verified the direct interaction between NR1H3 and NLRP3. NR1H3 not only binds to NLRP3's promoter, but also can interact with NLRP3's protein. Hence, NLRP3 signaling might be the downstream effector of NR1H3 signaling, and NLRP3 inflammasome participated in the mechanisms of NR1H3's protective roles in septic myocardial injury.

Physiological and pathological stimuli (such as ischemia, hypoxia, and poison), environmental factors, and nutritional imbalances lead to dysregulation of ER functions and accumulation of misfolded‐ or unfolded‐protein in ER, triggering ERS.^40^ In response to ERS, an intracellular signaling pathway called the unfolded protein response (UPR) restores ER homeostasis via activating PERK, IRE1, and ATF6.^41^ These molecules are bound to GRP78 respectively under normal conditions and remain inactive, while the separation of GRP78 from these molecules initiates the UPR. However, if ERS persists chronically at high levels, a terminal UPR program (overactivated PERK, IRE1, and ATF6) induces pathological cell apoptosis.^41^, ^42^ Studies have shown that the ERS‐related signaling pathway is widely involved in septic myocardial injury. Li et al. found that ERS‐related proteins (GRP78 and CHOP) and the apoptosis of myocardial cells were significantly increased after 12 h and 14 h of CLP injury in mice.^43^ Results of the study also showed that compared with the WT + CLP group, ATF6, CHOP, P‐PERK, PERK, and Bax protein levels were significantly increased in the heart of the NR1H3‐KO + CLP group. These results denote that the cardioprotection of NR1H3 is dependent on the regulation of ERS, at least partly.

During sepsis, myocardial tissues undergo tremendous oxidative stress, which contributes critically to heart dysfunction. The generation of ROS is one of the most deleterious reason of oxidative damage.^44^ The stimulation of endogenous antioxidant systems may protect cardiomyocytes against septic conditions.^45^, ^46^ Since oxidative stress is related to septic organ failure, antioxidant therapy may be a potential pharmacological treatment. In terms of the myocardial endogenous antioxidant, SIRT1 is a crucial target of endogenous antioxidants.^47^, ^48^, ^49^ In our *in vivo* model of sepsis, NR1H3 knockout significantly increased ROS release. However, we did not find that NR1H3 deficiency had a significant effect on the level of SIRT1. These findings illustrated that NR1H3 could reduce the excessive production of ROS induced by sepsis, while SIRT1 might not underlie the antioxidant stress effect of NR1H3.

Cellular energetic failure due to mitochondrial dysfunction is associated with worse outcomes for critically ill patients. Improvement of mitochondrial function is a protective tool to reduce cardiomyocyte susceptibility to stress.^50^ Mitochondrial biogenesis function is orchestrated by the several genes that control mitochondrial synthesis.^51^ PGC‐1α is considered as the master regulator of mitochondrial biogenesis by interacting with its downstream molecules, such as NRF1, TFAM, and UCP2.^52^ In addition, SIRT1 deacetylation of PGC‐1α implicates in metabolic control and mitochondrial biogenesis.^53^ PGC‐1α‐induced coordinated signaling is devoted to the control of mitochondrial biogenesis function homeostasis. In SH‐SY5Y cells, sitagliptin treatment activates PGC‐1α to up‐regulate mitochondrial NRF1, thus stimulating mitochondrial biogenesis and increasing mitochondrial mass.^54^ Similarly, the activated PGC‐1α/UCP2 pathway was also validated to be partially responsible for cerebral ischemia‐induced mitochondrial oxidative damage and mitochondria‐dependent apoptosis.^55^ Our data revealed that NR1H3 knockout decreased the protein levels of NRF1 and UCP2 of CLP mice, but had no remarkable changes in the expression of SIRT1 and PGC‐1αinsepticmice,suggeingthatNRF1 and UCP2might underline the mitochondrial function effect of NR1H3.

AMPK/ACC signaling pathway is a classic myocardial protection pathway that orchestrates the cellular response to stresses in the heart. AMPK plays an important role in myocardial fatty acid oxidation via phosphorylating ACC.^56^ AMPK activation by Sirtuin‐3 could improve myocardial mitochondrial biogenesis, which is required to sustain mitochondrial redox balance and suppress mitochondrial apoptosis during sepsis.^57^ In this study, NR1H3 deletion significantly down‐regulated the phosphorylation level of AMPK and ACC in septic mice, indicating that AMPK/ACC pathway might be involved in the regulatory mechanisms of NR1H3 in septic myocardial injury.

There are also some limitations in this study. First, as we all know, most endogenous and synthetic agonists, including T0901317, are no specific selectivity for the isoforms of LXRs. This means we cannot be entirely sure of the involvement of NR1H3 rather than NR1H2 in the cardioprotection of the LXRs agonist. Secondly, the differences in experimental settings, the severity of sepsis, and the dose of T0901317 may result in a contradiction to the previous research.^58^ Nonetheless, this study for the first time verified the potential beneficial effects of NR1H3 in the septic heart via constructing NR1H3 knockout mice in vivo.

## 4 CONCLUSIONS

**FIGURE 9:**
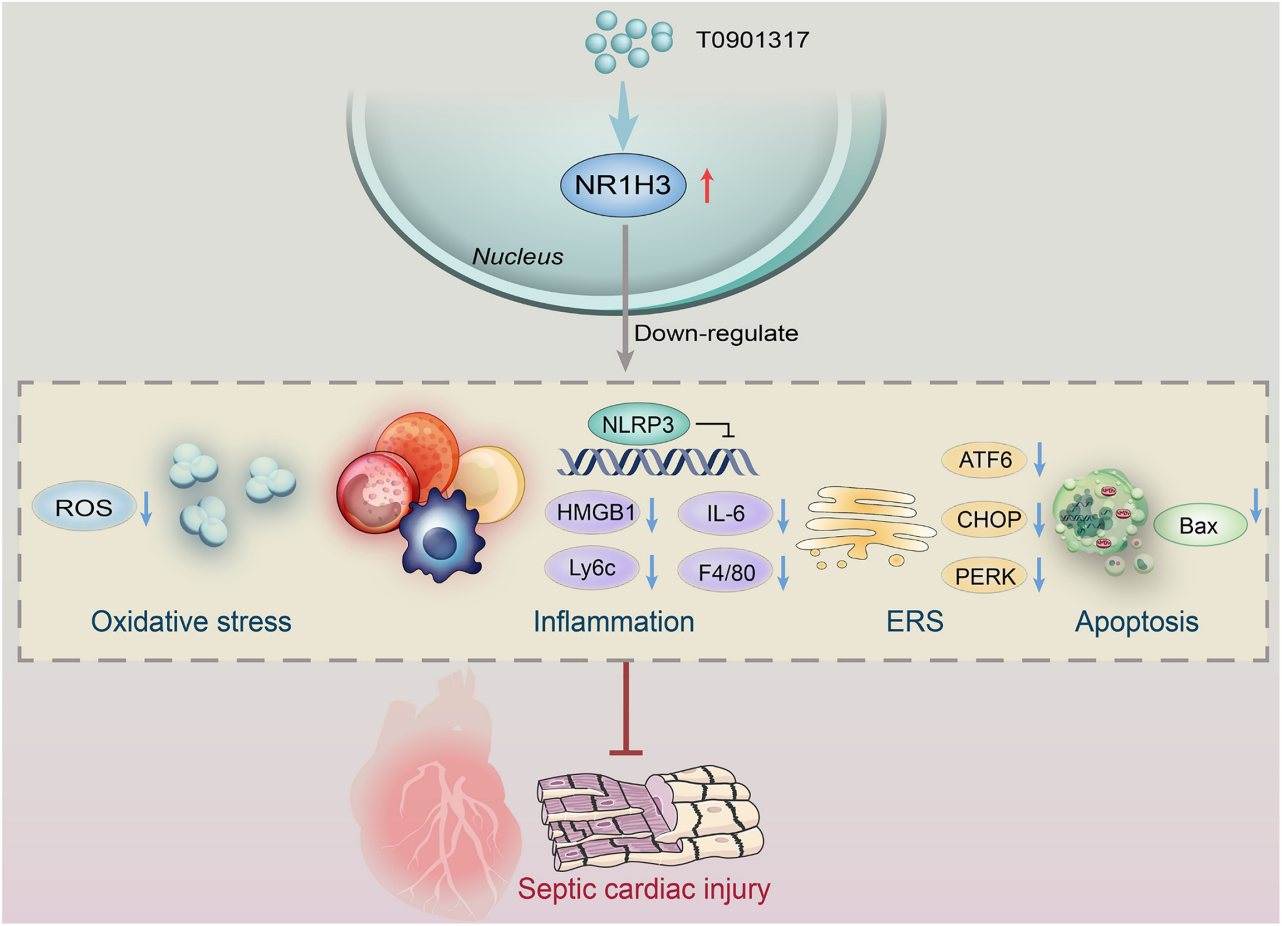
Proposed scheme depicting the mechanisms of NR1H3 protecting against sepsis‐induced heart failure via regulating NLRP3‐related signaling pathway.

In conclusion, the current study demonstrated a favorable role of the NR1H3‐mediated signalings in murine septic hearts, which primarily through attenuation of inflammation and oxidative stress, regulation of ERS, and repression of NLRP3 signaling pathway. In particular, NLRP3 serves as a downstream effector for NR1H3‐initiated myocardial benefits during sepsis (shown in Figure 9). Targeting NR1H3 may represent a promising approach for septic heart failure.

## 5 MATERIALS AND METHODS

### 5.1 Animals

All whole‐body knockout animals were identified by Southern blot and real‐time PCR analysis (Figure S2A,B). Balbc mice were used to investigate the effect of T0901317 on sepsis. Other experiments used C57BL/6 mice as wild type, Sham, or CLP group for the appropriate control. All mice had free access to food and water and were bred at 26°C in a 12 h light /12 h dark cycle.

### 5.2 Experimental animals

Study document that males are more prone than females to develop severe sepsis.^59^ In this study, male mice were performed in all animal experiments to minimize the effects of sex. Male C57BL/6 mice aged 8–10 weeks and weighed 22–25 g were obtained from the animal center of the Fourth Military Medical University (Xi'an, China). Adult male Balbc mice aged 8–10 weeks and weighed 22–25 g were obtained from the animal center of the Fourth Military Medical University (Xi'an, China). NR1H3 knockout (NR1H3−/−) mice (C57BL/6 background) were purchased from Kangweida Gene Technology Co., Ltd. (Wuhan, China). All animal experiment protocols were conducted in accordance with the guidelines of the Animal Care and Use Committees at Northwest University (Approval no. 2019026, Xi'an, Shaanxi, China). We made all efforts to minimize the number of animals used and to prevent their suffering.

### 5.3 CLP model

All mice were fasted for 8 h before the operation, but water was allowed ad libitum. Mice were anesthetized with 3% inhaled isoflurane. The mice's abdominal fur was shaved off, and the mice's abdominal skin was sterilized with 75% alcohol. A 1–2 cm midline laparotomy was performed to expose the cecum with the adjoining intestine. The cecum was tightly ligated at 1/3 site from its end using 4–0 nylon suture, and double punctures of the cecal wall were performed with a 25 G‐gauge needle. A small droplet of feces was squeezed through the puncture site to ensure patency, and it was returned to the peritoneal cavity. The incision of the peritoneum, fasciae, abdominal musculature, and skin was sutured with a sterile 6‐0 silk (which obtained a model with less injury, Figure S1A). For the survival experiment, an aggravated CLP model was constructed, which the cecum was tightly ligated at 2/3 site from its end (Figure S1D). Except for cecum ligation and perforation, the other operations in the sham operation group were the same as those in the operation group. All operated mice were resuscitated by injecting pre‐warmed normal saline (1 mL/100 g, 37°C) subcutaneously. Cardiac function was measured at post‐CLP 8 h or 24 h and then specimens were collected for further experiments. At 8 h after the surgery, cardiac function was assessed by echocardiography. Animals were euthanized at endpoint under isoflurane anesthesia followed by cervical dislocation, and hearts were removed for histological and molecular biological detection.

### 5.4 Quantitative real‐time PCR (qRT‐PCR)

Total RNA was extracted from cells using the TRIzol™ total RNA extraction kit (Takara Bio Inc., Kusatsu, Shiga, Japan), and reverse transcription was performed using the Prime Script RT Master Mix (Takara Bio Inc., Kusatsu, Shiga, Japan). Then the mRNA levels of NR1H3, NLRP3, ABC1A, ABCG1, PPARγ, IL‐6, IL‐1β, Caspase 1, and TNF‐α were detected using quantitative real‐time reverse transcriptase PCR analyses with SYBR Premix Ex Taq (Hunan Accurate Biotechnology Co. Ltd., Hunan, China). The detailed information primer sequence of qPCR is shown in Table S1. The reaction conditions were as follows: (1) 95°C for 10 min; (2) 40 cycles of 95°C for 5 s, 60°C for 30 s; (3) 94°C for 30 s, 60°C for 90 s, 94°C for 10 s. The expression levels of the examined transcripts were compared to that of β‐actin and normalized to the mean value of the controls.

### 5.5 Statistical analysis

Data were analyzed using GraphPad Prism 8 (LaJolla, CA, USA). All values are presented as the means ± standard deviations (SD). Sample sizes subjected to statistical analysis at least six animal per group. We selected the animal experimental groups with the smallest sample sizes as representatives for power analysis. When the significance level is 0.05 and the sample size is 6–7, the power is all more than 0.8, indicating that the conclusion of this study is reliable with the available sample size (Table S2). Significant differences were evaluated by Student's *t*‐test for comparing the means of two groups or one‐way ANOVA for multiple groups. When ANOVA indicated significance, multiple comparisons were performed using Tukey's HSD post hoc test. *p* < 0.05 indicated that the data were significantly different.

The more detailed information of methods are available in Supporting Information and a flowchart to precisely describe the methodology adopted in our study are shown in Figure S6.

## AUTHOR CONTRIBUTIONS

All authors have contributed significantly to this work and meet criteria for authorship by the International Committee of Medical Journal Editors recommendations. *Conception and design*: Yang Yang. *Acquisition of data*: Chao Deng, Qiong Liu, Huadong Zhao, Lu Qian, Wangrui Lei, Wenwen Yang, and Zhenxing Liang. *Analysis and interpretation of data*: All authors. *Drafting the manuscript for important intellectual content*: Chao Deng, Huadong Zhao, Qiong Liu, and Wangrui Lei. *Revising the manuscript for important intellectual content*: Zhenxing Liang, Ye Tian, Shaofei Zhang, Changyu Wang, and Ying Chen. Final approval of the version to be published: All authors.

## FUNDING INFORMATION

This work was supported by the National Natural Science Foundation of China (82070422 and 81871607), Youth Science and Technology Rising Star Project of Shaanxi Province (2020KJXX‐036), High‐end Foreign Expert Introduction Program of National Science and Technology (G2022040014L), and Innovation Capability Strong Foundation Plan of Xi'an City (Medical Research Project, 21YXYJ0037).

## CONFLICT OF INTEREST STATEMENT

The authors declare no conflicts of interest.

### PEER REVIEW

The peer review history for this article is available at https://www.webofscience.com/api/gateway/wos/peer-review/10.1002/btm2.10517.

## Supporting information


**Figure S1.** The establishment of mouse CLP models. (A) Photos indicating the protocol of mouse slight CLP models (1/3 ligation). (B) The sepsis score (*n* = 7 for Sham, *n* = 8 for CLP). (C) The anal temperature (*n* = 7 for each group). (D) The establishment of aggravated CLP models (2/3 ligation). Data are mean ± SD, *p < 0.05 versus the Sham group.
**Figure S2.** The NR1H3 knockout mice were constructed and confirmed by Southern blot and qPCR. (A) The Southern blot image of NR1H3 from WT and NR1H3 KO mice. (B) qPCR analysis of NR1H3 mRNA levels (*n* = 7 for each group). Data are mean ± SD. *p < 0.05 versus the WT group.
**Figure S3.** The effects of NR1H3 knockout on inflammatory response, ERS, apoptosis, oxidative stress, and mitochondrial function signaling pathways in CLP‐free mice. (A) Representative Western blot images of NR1H3, NLRP3, Caspase 1 p20, IL‐1β p32, IL‐1β p17, HMGB1, IL‐6, ATF6, GRP78, CHOP, P‐PERK, PERK, Bax, Bcl2, SIRT1, PGC‐1α, NRF1, UCP2, and TFAM in mouse heart tissues. (B) Quantitative analysis of these proteins in mouse heart tissues determined with GAPDH for normalization. *n* = 6 for each group. Data are mean ± SD, *p < 0.05 versus the WT group.
**Figure S4.** Additional echocardiographic data about NR1H3 knockout on cardiac function in septic mice. (A) LVPWd, LVPWs, LVEDV, and LVESV in the long‐axis view (*n* = 6 for each group). (B) LVPWd, LVPWs, LVEDV, and LVESV in the short‐axis view (*n* = 6 for each group). Data are mean ± SD, *p < 0.05 versus the WT + CLP group.
**Figure S5.** Nigerin reversed the protective role of T0901317 in HL‐1 cells stimulated by LPS. (A) Cell viability in HL‐1 cells treated with nigerin at different concentrations (2, 4, 6, 8, or 10 μM). (B) NLRP3 mRNA levels at different time points after nigerin addition in HL‐1 cells. (C) qPCR analysis of myocardial mRNA of NR1H3, NLRP3, IL‐6, IL‐1β, Caspase 1, and TNF‐α in HL‐1 cells. Data are expressed as the mean ± SD. *n* = 3, *p < 0.05 versus the Control group, ^#^ *p* < 0.05 versus the LPS group, and ^@^ *p* < 0.05 versus the T09 + LPS group.
**Figure S6.** A flowchart to describe the methodology adopted in our study. The workflow includes five parts: (1) The alteration of NR1H3 and NLRP3 in CLP mice was detected; (2) NR1H3 knockout mice with or without CLP were applied to elaborate the role of NR1H3 in septic myocardial injury; (3) T0901317 treatment *in vivo* or *in vitro* alleviated the septic injury, while nigerin exerted negative roles; (4) A series of methods were performed to explore the relationship between NR1H3 and NLRP3; (5) RNA‐sequencing identified transcriptome regulated by NR1H3 in septic mice.
**Figure S7.** Southern blot in luciferase reporter assays. (A) The result of NLRP3 promoters (2.2 kb, 1.7 kb, 1.2 kb, and 0.7 kb) by normal amplified PCR. (B) The result of double restriction enzyme digestion by SacI and XhoI. (C) The result of successfully constructed pGL3.basic vectors by PCR identification.
**Table S1.** The primer sequences of qPCR.
**Table S2.** Power analysis.

## Data Availability

The data that support the findings of this study are available from the corresponding author upon reasonable request. Some data may not be made available because of privacy or ethical restrictions.
