# Correction to “Activation of NR1H3 attenuates the severity of septic myocardial injury by inhibiting NLRP3 inflammasome”

**DOI:** 10.1002/btm2.10707

**Published:** 2024-08-15

Chao Deng , Qiong Liu , Huadong Zhao, Lu Qian, Wagnrui Lei , Wenwen Yang , Zhenxing Liang , Ye Tian , Shaofei Zhang , Changyu Wang , Ying Chen , Yang Yang . Activation of NR1H3 attenuates the severity of septic myocardial injury by inhibiting NLRP3 inflammasome. Bioeng Transl Med. 2023;8(3):e10517.37206244 10.1002/btm2.10517PMC10189481

An inaccuracy has been found in the statistical graph of ABCA1 in Figure 2c of the published article. The corrected version of Figure 2 is shown below.

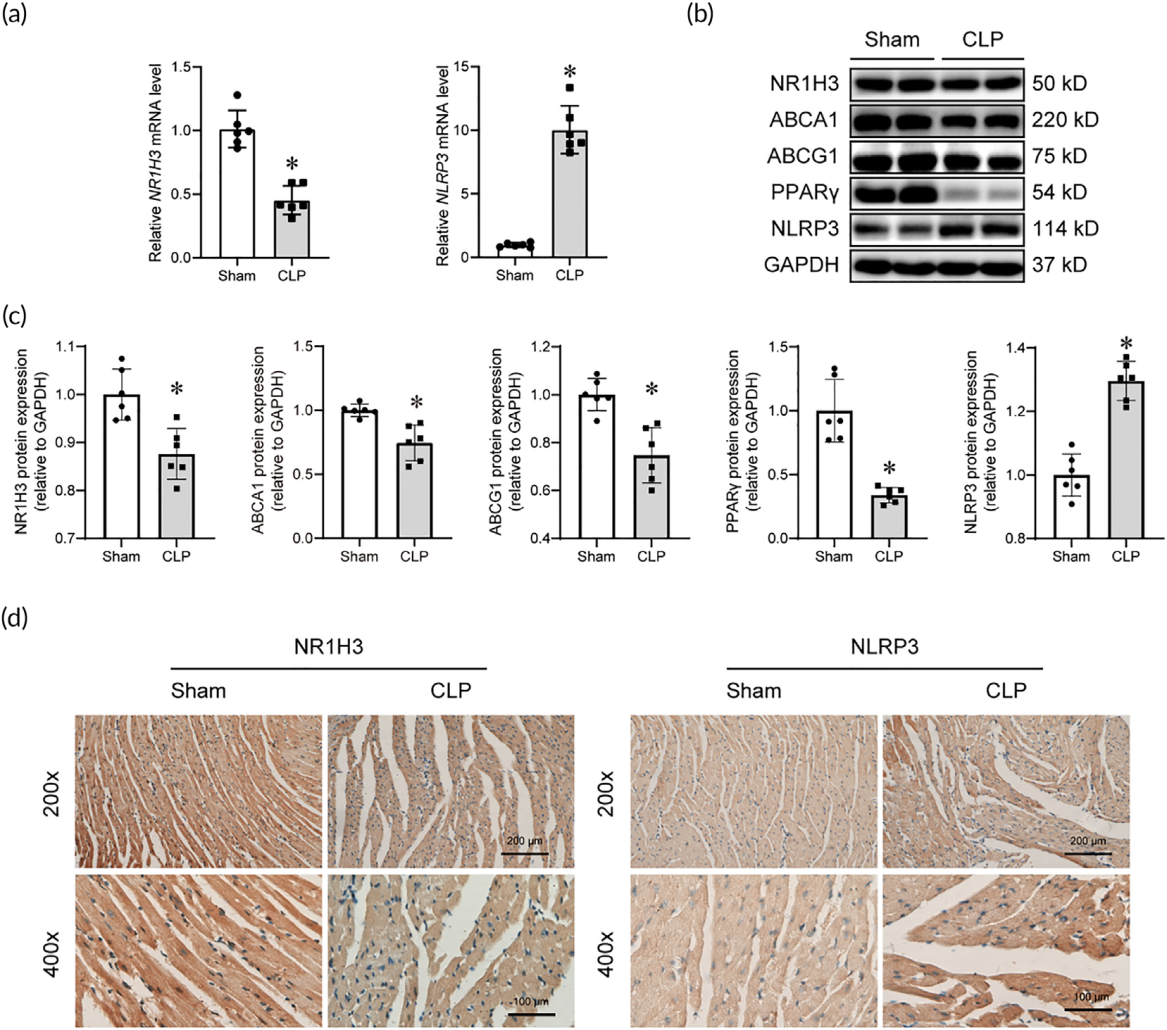

We apologize for this error.

