# Supplementary material for: Activation of NR1H3 attenuates the severity of septic myocardial injury by inhibiting NLRP3 inflammasome
Source: Bioeng Transl Med. 2023 Apr 6;8(3):e10517. doi: 10.1002/btm2.10517 (PMC10189481; doi:10.1002/btm2.10517)
Supplement: Supplementary file 1 — Figure S1. The establishment of mouse CLP models. (A) Photos indicating the protocol of mouse slight CLP models (1/3 ligation). (B) The sepsis score (n = 7 for Sham, n = 8 for CLP). (C) The anal temperature (n = 7 for each group). (D) The establishment of aggravated CLP models (2/3 ligation). Data are mean ± SD, *p < 0.05 versus the Sham group. Figure S2. The NR1H3 knockout mice were constructed and confirmed by Southern blot and qPCR. (A) The Southern blot image of NR1H3 from WT and NR1H3 KO mice. (B) qPCR analysis of NR1H3 mRNA levels (n = 7 for each group). Data are mean ± SD. *p < 0.05 versus the WT group. Figure S3. The effects of NR1H3 knockout on inflammatory response, ERS, apoptosis, oxidative stress, and mitochondrial function signaling pathways in CLP‐free mice. (A) Representative Western blot images of NR1H3, NLRP3, Caspase 1 p20, IL‐1β p32, IL‐1β p17, HMGB1, IL‐6, ATF6, GRP78, CHOP, P‐PERK, PERK, Bax, Bcl2, SIRT1, PGC‐1α, NRF1, UCP2, and TFAM in mouse heart tissues. (B) Quantitative analysis of these proteins in mouse heart tissues determined with GAPDH for normalization. n = 6 for each group. Data are mean ± SD, *p < 0.05 versus the WT group. Figure S4. Additional echocardiographic data about NR1H3 knockout on cardiac function in septic mice. (A) LVPWd, LVPWs, LVEDV, and LVESV in the long‐axis view (n = 6 for each group). (B) LVPWd, LVPWs, LVEDV, and LVESV in the short‐axis view (n = 6 for each group). Data are mean ± SD, *p < 0.05 versus the WT + CLP group. Figure S5. Nigerin reversed the protective role of T0901317 in HL‐1 cells stimulated by LPS. (A) Cell viability in HL‐1 cells treated with nigerin at different concentrations (2, 4, 6, 8, or 10 μM). (B) NLRP3 mRNA levels at different time points after nigerin addition in HL‐1 cells. (C) qPCR analysis of myocardial mRNA of NR1H3, NLRP3, IL‐6, IL‐1β, Caspase 1, and TNF‐α in HL‐1 cells. Data are expressed as the mean ± SD. n = 3, *p < 0.05 versus the Control group, # p < 0.05 versus the LPS group, and [file BTM2-8-e10517-s001.docx]

**SUPPLEMENTARY METHODS**

**Survival assessment and sepsis score**

For the survival assessment during 72 h, an aggravated CLP model was constructed. To be specific, the cecum was tightly ligated at 2/3 site from its end (Supplementary Figure 2d); the other operations were the same as described above. Disease severity were determined at 8 h post-CLP with a digital thermometer and a scoring system, murine sepsis score (MSS), developed by Shrum et al ^1^ The MSS criteria include the degree of piloerection, spontaneous activity, response to stimuli, level of consciousness, the openness of eyes, and the posture and degree of labored breathing. Each criterion was scored from 0 to 4, and a total MSS score was calculated. Mortality rises as MSS increases. The anal temperature was detected at post-CLP 8 h with the animal thermometer (Calvin Biotechnology Co., Ltd., Nanjing, Jiangsu, China).

**Cell lipopolysaccharide (LPS) model**

Murine HL-1 cells obtained from American Type Culture Collection (ATCC, Rockville, MD, USA) were maintained in Dulbecco’s Modified Eagle Medium (DMEM) containing 10% fetal bovine serum (FBS) and incubated at 37 ℃ under 5% CO_2_. When HL-1 cells grew to 80% confluent in 60 mm culture dishes, the medium was replaced with a fresh serum-free medium. Finally, the HL-1 cells were exposed to 10 ug/ml LPS for 6 h.

**T0901317 Treatment**

T0901317 was dissolved in dimethyl sulfoxide (DMSO). For *in vitro* experiments, the final concentration of DMSO in the cell culture studies was 0.5% (v/v) or less. The control group was treated with FBS-free DMEM containing DMSO at equal volumes. The model groups were treated with 10 μM T0901317 or vehicle for 6 h, followed by the addition of LPS. For the animal experiment, T0901317 (10 mg/kg, i.p.) was administrated every 2 days for 6 days before CLP challenge.

**Cell viability assay**

Cells were digested with trypsin and supernatant was discarded following centrifugation. Cells were prepared into single cell suspension using PBS. Cell suspension was with Muse^TM^ Count &Viability Assay Kit (MCH100104, Merck&Millipore, Germany) for 5 minutes in the dark at room temperature. Cell viability was measured using the Muse^TM^ cell analyzer (Merck&Millipore, Germany).

**Apoptosis assay**

A Muse^TM^ Cell Analyzer was used according to the manufacturer’s recommendations. Briefly, cells were harvested and were stained with a Muse^TM^ Annexin V & Dead Cell Kit (MCH100105, Merck&Millipore, Germany).

**Detection of blood biochemical parameters**

At 8 h post-CLP, 10 μL blood was collected from the left eyeball in a strictly sterile procedure. 150 μL serum was then collected from the blood. The levels of lactate dehydrogenase (LDH), creatine kinase (CK), alanine aminotransferase (ALT), aspartate aminotransferase (AST), blood urea nitrogen (BUN), and albumin (ALB) were detected by an automatic Blood biochemistry analyzer (XinRui Technology Co., Ltd, XR210, Zhongshan, Guangdong, China).

**Echocardiography evaluation**

Transthoracic echocardiography was performed using an animal-specific instrument (VisualSonics Vevo3100, VisualSonics, Toronto, ON, Canada) at 8 h post-CLP in all animals. Anesthesia was induced with 3% isoflurane and 1 L/min 100% oxygen in an induction chamber for 1-2 min. Once the animal lost its righting reflex, it was laid supine on a warm platform with its nose enveloped in a nose cone to keep the mouse anesthetized by 2% isoflurane, and M-mode images were recorded. Echocardiographic images were recorded in a digital format. The cardiac function parameters, including cardiac output (CO), stroke volume (SV), left ventricular diastolic volume (LVEDV), left ventricular systolic volume (LVESV), left ventricular end-diastolic posterior wall thickness (LVPWd), left ventricular end-systolic posterior wall thickness (LVPWs), heart rate (HR), left ventricular diastolic volume (LVEDV), left ventricular systolic volume (LVESV) were calculated using computer algorithms. All measurements were based on 3 consecutive cardiac cycles.

**Histological staining**

The myocardium was fixed in 4% paraformaldehyde and sectioned at a thickness of 4-5 µm. Morphological changes in myocardial tissues were observed by hematoxylin-eosin (H&E) staining. Myocardial reactive oxygen species generation was examined by dihydroethidium (DHE) staining (Beyotime Biotechnology, Shanghai, China). For immunostaining, paraffin-embedded slices were stained with the respective primary antibodies against NR1H3 (1:200; Bioss Biotechnology Co., Ltd, Beijing, China), NLRP3 (1:200; Bioss Biotechnology Co., Ltd, Beijing, China), IL-1β (1:200; Bioss Biotechnology Co., Ltd, Beijing, China), MPO (1:200; Servicebio, Wuhan, China), Ly6c and F4/80 (1:200; Santa Cruz Biotechnology, Dallas, TX, USA), CD59a (1:200; Boster Biological Technology Co., Ltd, CA, USA) then incubated with a secondary biotinylated anti-rabbit IgG, stained with 3,3’-diaminobenzidine (DAB), and imaged using a microscope (Invitrogen EVOS M5000, Thermo Fisher Scientific, Waltham, MA, USA).

**Southern blot**

The 0.5-1.0 cm tail of the mouse was cut into a DNA enzyme-free centrifuge tube under a sterile environment. Then, 98 μL Lysis Buffer and 2 μL Proteinase K (Beyotime Biotechnology, Shanghai, China) were added. The mixture was centrifuged for 10 s and digested in a 55 ℃ water bath overnight. The next day, the lysate was boiled at 100 ℃ for 5 min and then centrifuged for 1 min. PCR amplification was performed using the 2× Taq Master Mix (Vazyme Biotech Co., Ltd, Nanjing, China). 0.5 g agarose gel and 50 mL TAE (Sangon Biotech, Shanghai, China) solution were boiled in the microwave for 2 min, and 5 μL nucleic acid dye (Beyotime Biotechnology, Shanghai, China) was added. Then, the solvent gel was poured into the gel plate. After 15 minutes, the DNA marker (Beyotime Biotechnology, Shanghai, China) and DNA amplified samples were labeled (90 V, 30 min) and imaged with a gel imager (JUNYI Electrophoresis Co., Ltd., Beijing, China).

**Luciferase reporter assay**

Cells were transfected with 500 ng of luciferase reporter plasmids, 50 ng of pGL3.basic vector (an internal control with renilla luciferase gene, Promega 2800 Woods Hollow Road · Madison, USA). The transfection assays were performed using Lipo2000 (Invitrogen, Life Technologies, Carlsbad, CA) according to the manufacturer’s protocol. The next day, the medium was changed to FBS-free Dulbecco’s modiﬁed Eagle medium with or without T0901317. Luciferase activities were determined by the Dual-Luciferase Reporter Assay System and GloMaxTM 20/20 luminometer (Promega). DNA agarose gel electrophoresis results showed that the bands of NLRP3 promoters (2.2 kb, 1.7 kb, 1.2 kb, 0.7 kb) were within the expected range. The amplification products of NLRP3 promoters were successfully obtained and can be purified with a DNA purification kit for the next experiments (Figure S7A). Then, we analyzed the double restriction enzyme site by BioXM.2.6 and pGL3.basic plasmid map and selected SacI and XhoI as specific restriction enzymes at last. DNA agarose gel electrophoresis results showed that the bands of restriction enzyme digestion of NLRP3 promoters (2.2 kb, 1.7 kb, 1.2 kb, and 0.7 kb) and pGL3.basic were within the expected range, which can be ligated by T4 DNA Ligase (Wyman Street, Waltham, MA, USA) (Figure S7B). DNA agarose gel electrophoresis results showed that NLRP3 promoters (2.2 kb, 1.7 kb, 1.2 kb, and 0.7 kb) had been successfully constructed on pGL3.basic. The positive cloned strain was selected and sent to Shanghai Sangon Biotechnology Limited Company for sequencing (Figure S7C). The sequencing results of NLRP3 promoters 2.2 kb, 1.7 kb, 1.2 kb, and 0.7 kb were compared with DNAMAN8 software. The relative luciferase activity values of treated cells were normalized to that of control cells.

**Western blot**

Isolated heart tissue from mice was homogenized in RIPA buffer containing protease and phosphatase inhibitors (Beyotime Biotechnology, Shanghai, China). The protein concentration was assessed by Enhanced BCA Protein Assay Kit (Beyotime Biotechnology, Shanghai, China). 30-50 μg of total protein extract was applied to 10% (or 8%) SDS-PAGE and transferred onto PVDF membranes. The membranes were blocked with 5% defatted milk and incubated with antibodies against NR1H3, ABCG1 (Abcam, Cambridge, United Kingdom), ABCA1, IL-6, Bax, NRF1, Nrf2, Ly6c (Boster Biological Technology co.ltd, Inc.USA), p-ACC, ACC, GRP78, CHOP, PERK (Cell Signaling Technology, Inc, USA), NLRP3, Caspase 1, F4/80 (Servicebio, Wuhan, China), IL-1β, SIRT1, p-PERK (Bioss Biotechnology Co., Ltd, Beijing, China), PPARγ, TFAM, NQO1, HO-1, ATF6 (Santa Cruz Biotechnology, Dallas, TX, USA), and GAPDH, HMGB1, UCP2, PGC-1α (Servicebio, Wuhan, China), Bcl2, AMPK (Abcam, Cambridge, United Kingdom), p-AMPK (Bimake, Houston TX, USA), and CD59a (Immunoway, Suzhou, Jiangsu, China). The fluorescent signal was detected using a MiNiChemi610 imaging system (SAGECREATION Co., Ltd, Beijing, China), and the signal was quantified using ImageJ software (National Institutes of Health, Bethesda, MD, USA).

**Co-immunoprecipitation**

Total proteins of isolated heart tissues were extracted using IP lysis buffer (Beyotime Biotechnology, Shanghai, China). Anti-NLRP3, anti-NR1H3 (Santa Cruz Biotechnology, Dallas, TX, USA), and IgG antibody (Beyotime Biotechnology, Shanghai, China) were incubated with cell lysate overnight at 4 ℃. Antibody-protein conjugates were pulled down by incubation with protein A/G agarose beads (Beyotime Biotechnology, Shanghai, China) for 4 h. Finally, the beads were washed, boiled, centrifuged. SDS-PAGE separated the recovered samples for Western blot analysis described above.

**Chromatin immunoprecipitation (ChIP)**

ChIP analysis was conducted with a ChIP Assay Kit (Beyotime Biotechnology, Shanghai, China) following the manufacturer’s instructions. In brief, 1*10^6^ cells were cross-linked by adding formaldehyde directly to cell culture media and incubated for 10min at 37 ℃, and then glycine was added to terminate the reaction. The fixed cells were washed with 10 mL ice-cold phosphate buffered saline twice and cells were scraped and resuspended in 400 μL SDS lysis buffer. The cell lysates were centrifuged and resuspended before being sonicated to generate 200-1,000 bp fragments. Chromatin extracts were diluted 10-fold in dilution buffer and preincubated for 30 min at 4 ℃ with 70 μL Salmon Sperm DNA/protein A-agarose. 50 μL diluted supernatant was kept for isolation of input DNA. After pelleting the agarose by brief centrifugation, 1μg of anti-NR1H3 (Santa Cruz Biotechnology, Dallas, TX, USA) antibody or anti-IgG (Beyotime Biotechnology, Shanghai, China) was added to the supernatant fraction and incubated overnight at 4 ℃ with rotation. In addition, the supernatant fraction with Salmon Sperm DNA/protein A-agarose was incubated for 60min at 4℃. 60μL of Salmon Sperm DNA/protein A-agarose was then added, and the mixture was incubated for 60min at 4℃ to collect the antibody/antigen–DNA complex. The chromatin bound to the protein A-agarose beads was eluted in 500μL of freshly prepared elution buffer (1% SDS, 0.1M NaHCO_3_). After reversing the cross-linking, the DNA was purified and recovered by Gel Extraction Kit (Omega Biotechnology, Guangzhou, China), and Pellets were resuspended in 30 μL of ddH_2_O for later use. Coprecipitated chromatin was analyzed by PCR for the presence of *NLRP3* promoter.

**mRNA sequencing by Illumina HiSeq**

The sequencing results were provided by NovelBio Technology Co., Ltd. (Shanghai, China). Total RNA of each sample was extracted using Trizol Reagent (Invitrogen)/RNeasy Mini Kit (Qiagen)/other kits. Total RNA of each sample was quantified and qualified by Agilent 2100 Bioanalyzer (Agilent Technologies, Palo Alto, CA, USA), NanoDrop (Thermo Fisher Scientific Inc.), and 1% agarose gel. 1 μg total RNA with RIN value above 6.5 was used for the following library preparation. Next-generation sequencing library preparations were constructed according to the manufacturer’s protocol. The poly(A) mRNA isolation was performed using Poly(A) mRNA Magnetic Isolation Module or rRNA removal Kit. The mRNA fragmentation and priming were performed using First Strand Synthesis Reaction Buffer and Random Primers. First strand cDNA was synthesized using ProtoScript II Reverse Transcriptase, and the second-strand cDNA was synthesized using Second Strand Synthesis Enzyme Mix. The purified double-stranded cDNA by beads was then treated with End Prep Enzyme Mix to repair both ends and add a dA- tailing in one reaction, followed by a T-A ligation to add adaptors to both ends. The size selection of Adaptor-ligated DNA was then performed using beads, and fragments of ~420 bp (with the approximate insert size of 300 bp) were recovered. PCR then amplified each sample for 13 cycles using P5 and P7 primers, with both primers carrying sequences which can anneal with flow cell to perform bridge PCR and P7 primer carrying a six-base index allowing for multiplexing. The PCR products were cleaned up using beads, validated using a Qsep100 (Bioptic, Taiwan, China), and quantified by a Qubit3.0 Fluorometer (Invitrogen, Carlsbad, CA, USA). Then libraries with different indices were multiplexed and loaded on an Illumina HiSeq instrument according to the manufacture’s instructions (Illumina, San Diego, CA, USA). Sequencing was carried out using a 2x150bp paired-end (PE) configuration; image analysis and base calling were conducted by the HiSeq Control Software (HCS) + OLB + GAPipeline-1.6 (Illumina) on the HiSeq instrument. The sequences were processed and analyzed by GENEWIZ.

**Molecular docking**

The protein structures of NR1H3 (PDB ID: 3IPQ) and NLRP3 (PDB ID: 7ALV) were obtained from PDB database. The equilibrium coefficient model of ClUSPro V2.0 (https://cluspro.org/) was adopted, and other parameters were default. According to the model scores of balance coefficient of 3IPQ and 7ALV, the model group with the largest cluster size (the most member values) was selected. Protein binding complex was additionally predicted by ZDOCK SERVER ^2^ and binding energy was calculated using PDBePISA ^3^. The docking results were visualized by Discovery Studio V2019.

**Bioinformatics analysis**

**Quality control**

In order to remove technical sequences, including adapters, PCR primers, or fragments thereof, and quality of bases lower than 20, pass filter data of fastq format were processed by Cutadapt (V1.9.1) to be high-quality clean data. *Principal component analysis.* Principal component analysis is another way to visualize sample-to-sample distances. In this ordination method, the data points (here, the samples) are projected onto the 2D plane such that they spread out in the two directions that explain most of the differences. The x-axis is the direction that separates the data points the most. The values of the samples in this direction are written PC1. The y-axis is a direction (it must be orthogonal to the first direction) that separates the data the second most. The values of the samples in this direction are written PC2. The percent of the total variance that is contained in the direction is printed in the axis label. These percentages do not add to 100% because more dimensions contain the remaining variance (although each of these remaining dimensions will explain less than the two that we see). This analysis has been dealt with the R language.

**Mapping**

Firstly, reference genome sequences and gene model annotation files of relative species were downloaded from the genome website, such as UCSC, NCBI, ENSEMBL. Secondly, Hisat2 (v2.0.1) was used to index the reference genome sequence. Finally, clean data were aligned to the reference genome via software Hisat2 (v2.0.1).

**Expression analysis**

In the beginning, transcripts in fasta format are converted from known gff annotation file and indexed properly. Then, with the file as a reference gene file, HTSeq (v0.6.1) estimated gene and isoform expression levels from the pair-end clean data.

**Differential expression analysis.**

Differential expression analysis used the DESeq2 Bioconductor package, a model based on the negative binomial distribution. The estimates of dispersion and logarithmic fold changes incorporate data-driven prior distributions, Padj of genes were set <0.05 to detect differential expressed ones. **Gene Ontology (GO) and Kyoto Encyclopedia of Genes and Genomes (KEGG) enrichment analysis**

GO-Seq (v1.34.1) was used to identify GO terms that annotate a list of enriched genes with a significant padj less than 0.05. Moreover, top-GO was used to plot DAG. KEGG is a collection of databases dealing with genomes, biological pathways, diseases, drugs, and chemical substances (http://en.wikipedia.org/wiki/KEGG). We used scripts in house to enrich significant differential expression genes in KEGG pathways.

**SUPPLEMENTARY TABLES**

**Supplementary Table 1** The primer sequences of qPCR.

| Name | Sequence (5’-3’) |
| --- | --- |
| NR1H3-F | 5’-ATCGCCTTGCTGAAGACCTCTG-3’ |
| NR1H3-R | 5’-CTGCTTTGGCAAAGTCTTCCCG-3’ |
| NLRP3-F | 5’-TCACAACTCGCCCAAGGAGGAA-3’ |
| NLRP3-R | 5’-AAGAGACCACGGCAGAAGCTAG-3’ |
| ABCA1-F | 5’-GGAGCCTTTGTGGAACTCTTCC-3’ |
| ABCA1-R | 5’-CGCTCTCTTCAGCCACTTTGAG-3’ |
| ABCG1-F | 5’-GACACCGATGTGAACCCGTTTC-3’ |
| ABCG1-R | 5’-GCATGATGCTGAGGAAGGTCCT-3’ |
| PPARγ-F | 5’-GTACTGTCGGTTTCAGAAGTGCC-3’ |
| PPARγ-R | 5’-ATCTCCGCCAACAGCTTCTCCT-3’ |
| IL-6-F | 5’-TACCACTTCACAAGTCGGAGGC-3’ |
| IL-6-R | 5’-CTGCAAGTGCATCATCGTTGTT-3’ |
| IL-1β-F | 5’-TGGACCTTCCAGGATGAGGACA-3’ |
| IL-1β-R | 5’-GTTCATCTCGGAGCCTGTAGTG-3’ |
| Caspase 1-F | 5’-GGCACATTTCCAGGACTGACTG-3’ |
| Caspase 1-R | 5’-GCAAGACGTGTACGAGTGGTTG-3’ |
| TNF-α-F | 5’-GGTGCCTATGTCTCAGCCTCTT-3’ |
| TNF-α-R | 5’-GCCATAGAACTGATGAGAGGGAG-3’ |

**Supplementary Table 2 Power analysis**

| **Group1** | **Group2** | **Group3** | **d** | **n** | **Power** |
| --- | --- | --- | --- | --- | --- |
| Sham | CLP | - | 2.1372243 | 7 | 0.96 |
| 616.5 | 1080.6 | - |  |  |  |
| 466.7 | 1143.6 | - |  |  |  |
| 995.8 | 1966.5 | - |  |  |  |
| 1030.5 | 1668.6 | - |  |  |  |
| 1040.7 | 2265 | - |  |  |  |
| 985.7 | 1604.4 | - |  |  |  |
| 1098.6 | 1647.6 | - |  |  |  |
| WT | NR1H3 KO | - | 2.6463954 | 6 | 0.98 |
| 0.906544 | 0.684491 | - |  |  |  |
| 0.909496 | 0.279107 | - |  |  |  |
| 0.876268 | 0.144892 | - |  |  |  |
| 1.093862 | 0.861153 | - |  |  |  |
| 1.125044 | 0.365109 | - |  |  |  |
| 1.088786 | 0.198672 | - |  |  |  |
| **Group1** | **Group2** | **Group3** | **f** | **n** | **Power** |
| Sham | CLP | T0901317+CLP | 1.6438 | 6 | 0.99 |
| 13.43218 | 6.757154 | 7.491996 |  |  |  |
| 13.70446 | 3.875219 | 13.16467 |  |  |  |
| 12.37634 | 7.092081 | 13.87241 |  |  |  |
| 14.54267 | 3.240361 | 11.3062 |  |  |  |
| 13.6442 | 6.415509 | 6.473051 |  |  |  |
| 12.82569 | 5.377207 | 10.46642 |  |  |  |

**SUPPLEMENTARY FIGURES**


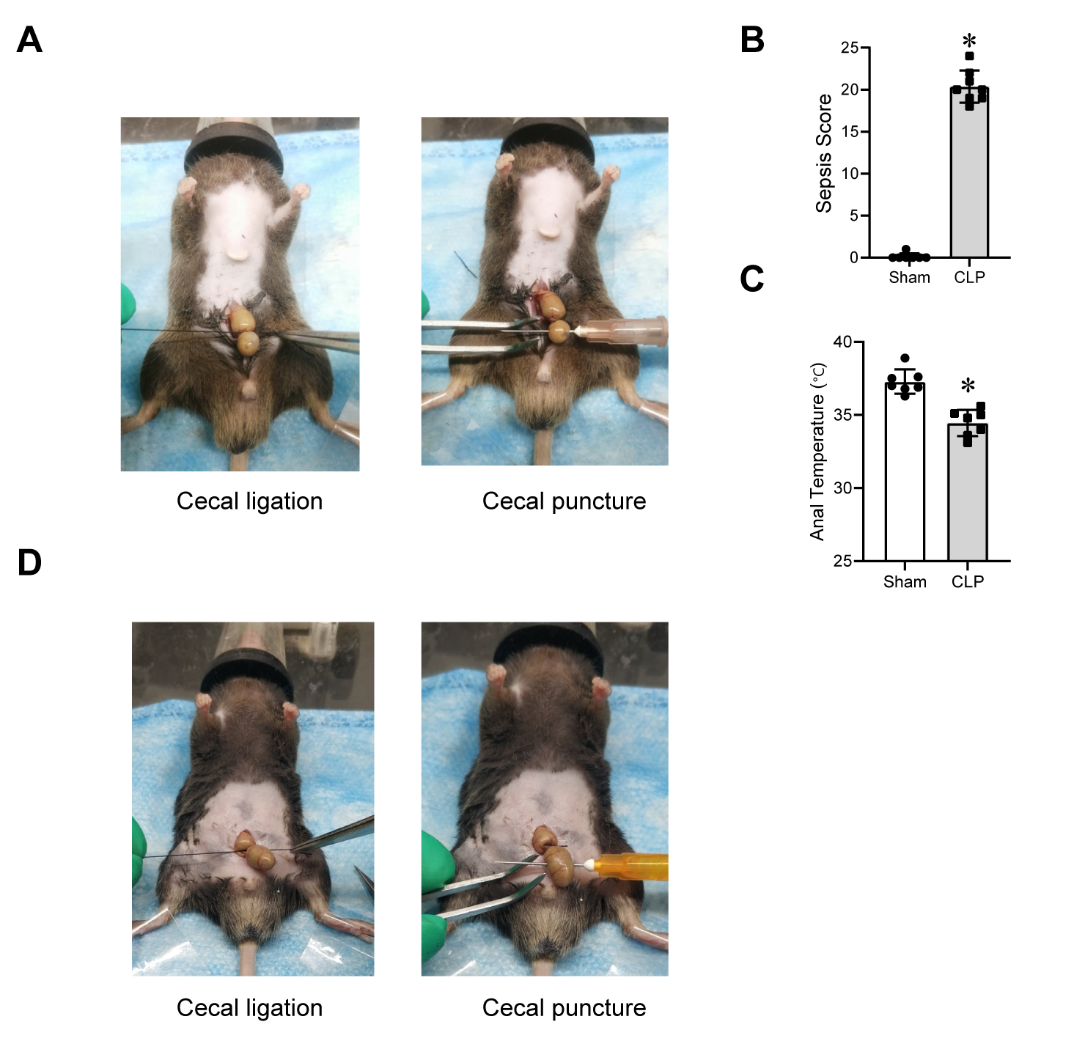


**FIGURE S1 The establishment of mouse CLP models.** **(A)** Photos indicating the protocol of mouse slight CLP models (1/3 ligation). **(B)** The sepsis score (n=7 for Sham, n=8 for CLP). **(C)** The anal temperature (n=7 for each group). **(D)** The establishment of aggravated CLP models (2/3 ligation). Data are mean±SD, **P*<0.05 vs. the Sham group.


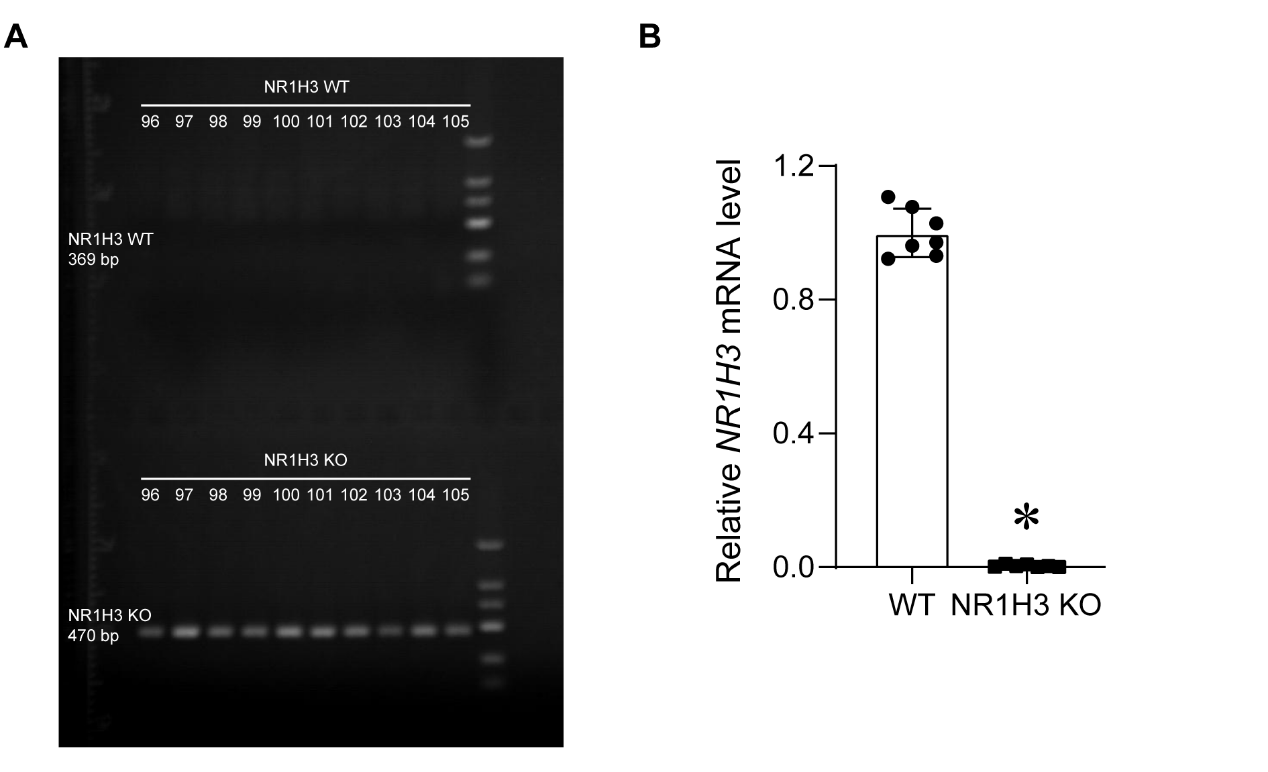


**FIGURE S2 The NR1H3 knockout mice were constructed and confirmed by Southern blot and qPCR. (A)** The Southern blot image of NR1H3 from WT and NR1H3 KO mice. **(B)** qPCR analysis of NR1H3 mRNA levels (n=7 for each group). Data are mean±SD. **P*<0.05 vs. the WT group.


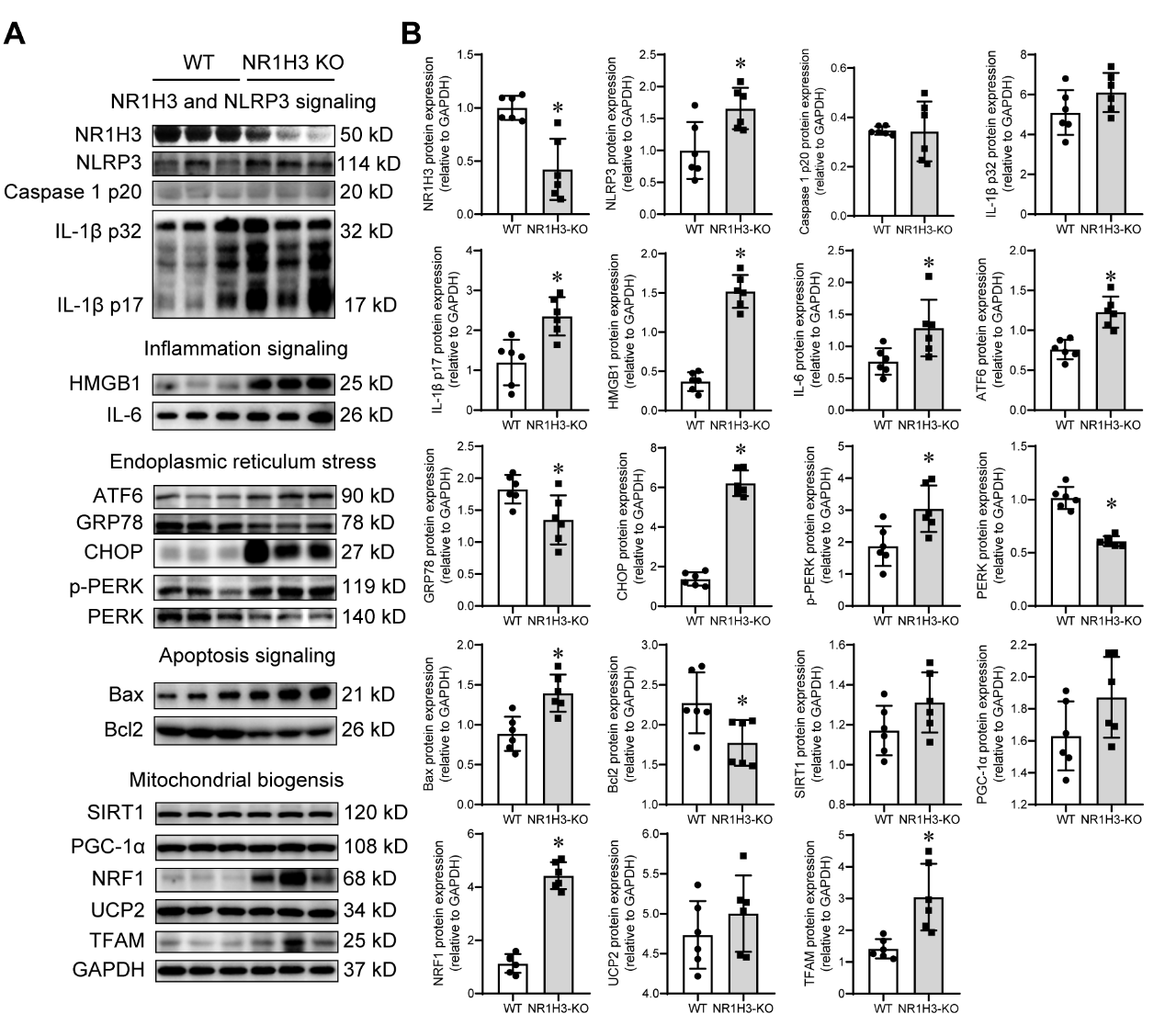


**FIGURE S3 The effects of NR1H3 knockout on inflammatory response, ERS, apoptosis, oxidative stress, and mitochondrial function signaling pathways in CLP-free mice.** **(A)** Representative Western blot images of NR1H3, NLRP3, Caspase 1 p20, IL-1β p32, IL-1β p17, HMGB1, IL-6, ATF6, GRP78, CHOP, P-PERK, PERK, Bax, Bcl2, SIRT1, PGC-1α, NRF1, UCP2, and TFAM in mouse heart tissues. **(B)** Quantitative analysis of these proteins in mouse heart tissues determined with GAPDH for normalization. n=6 for each group. Data are mean±SD, **P*<0.05 vs. the WT group.


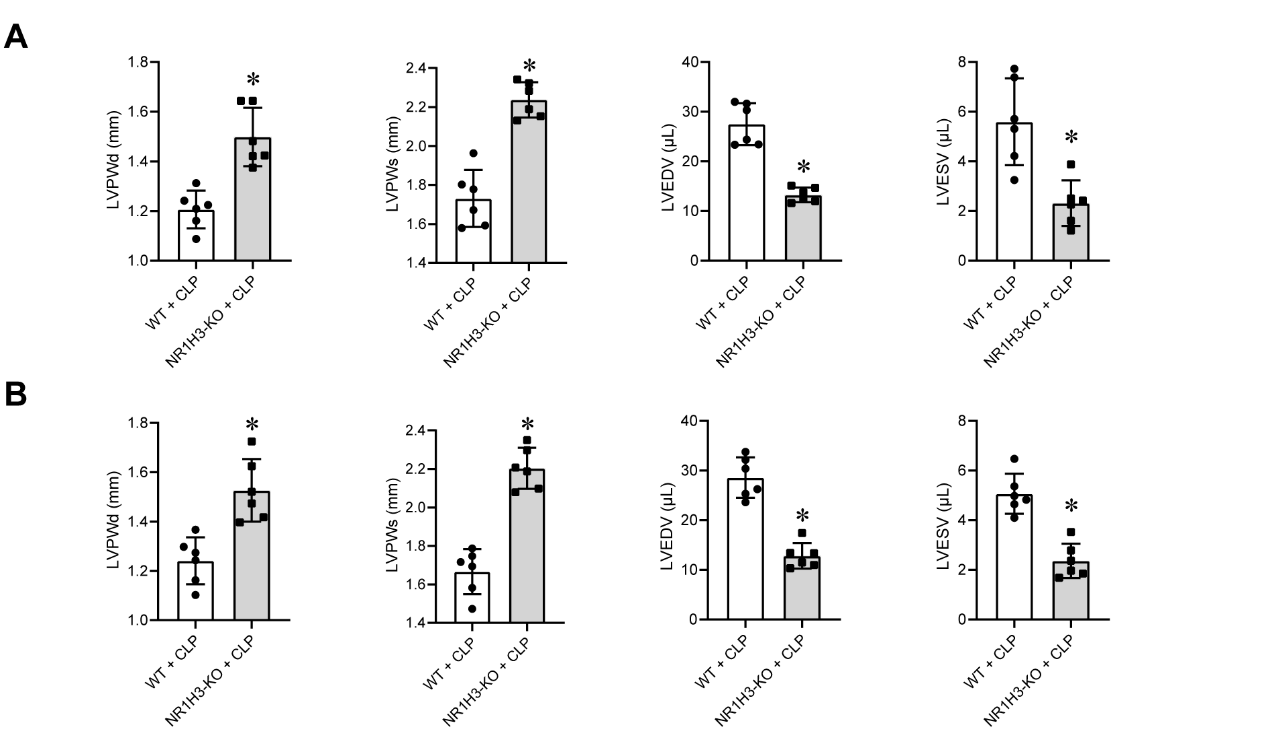


**FIGURE S4 Additional echocardiographic data about NR1H3 knockout on cardiac function in septic mice. (A)** LVPWd, LVPWs, LVEDV, and LVESV in the long axis view (n=6 for each group). **(B)** LVPWd, LVPWs, LVEDV, and LVESV in the short axis view (n=6 for each group). Data are mean±SD, **P*<0.05 vs. the WT+CLP group.


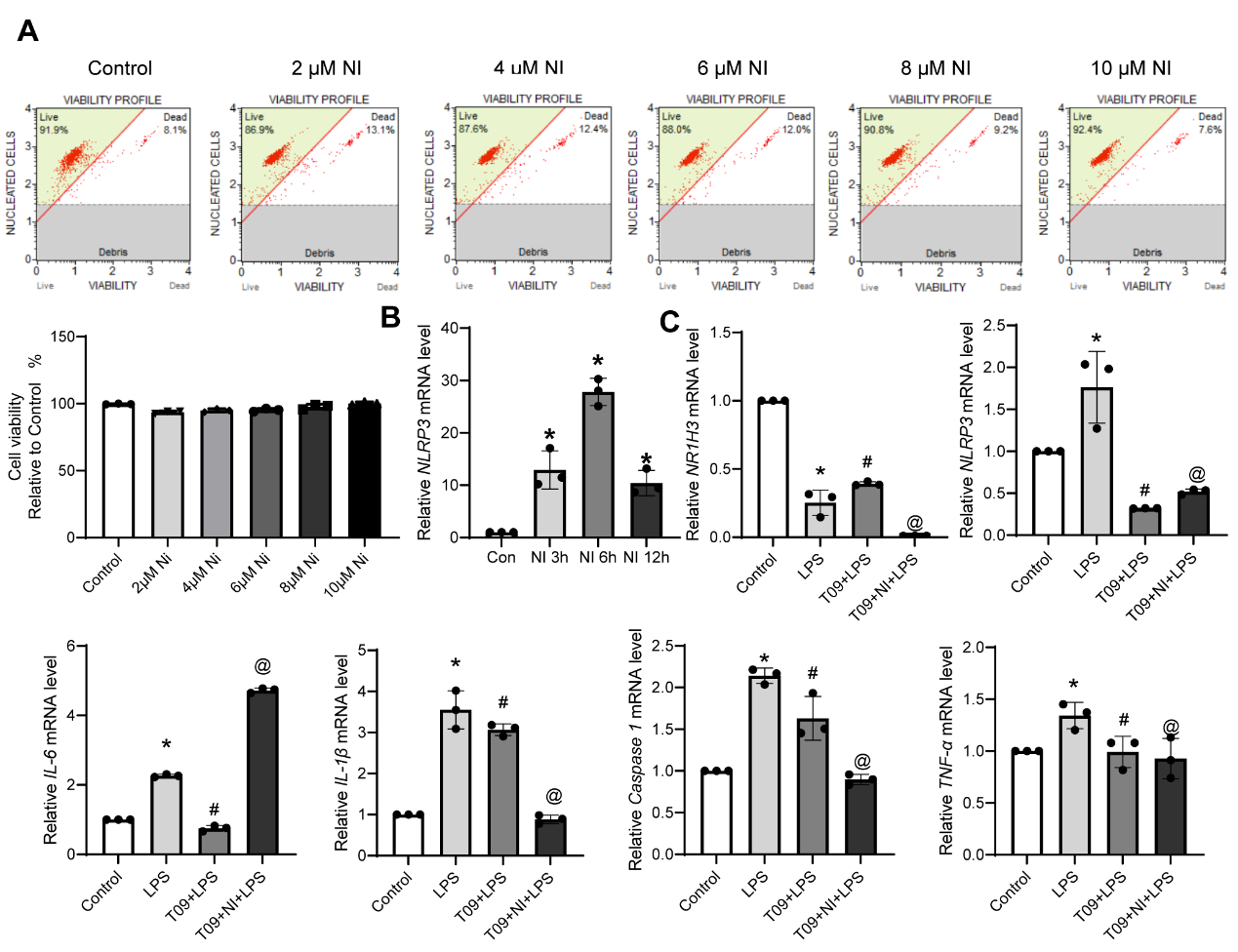


**FIGURE S5 Nigerin reversed the protective role of T0901317 in HL-1 cells stimulated by LPS. (A)** Cell viability in HL-1 cells treated with nigerin at different concentrations (2, 4, 6, 8, or 10 μM) **(B)** NLRP3 mRNA levels at different time points after nigerin addition in HL-1 cells. (**C)** qPCR analysis of myocardial mRNA of NR1H3, NLRP3, IL-6, IL-1β, Caspase 1, and TNF-α in HL-1 cells. Data are expressed as the mean±SD. **P*<0.05 vs. the Control group, ^#^*P*<0.05 vs. the LPS group, and ^@^*P*<0.05 vs. the T09+LPS group. n=3.


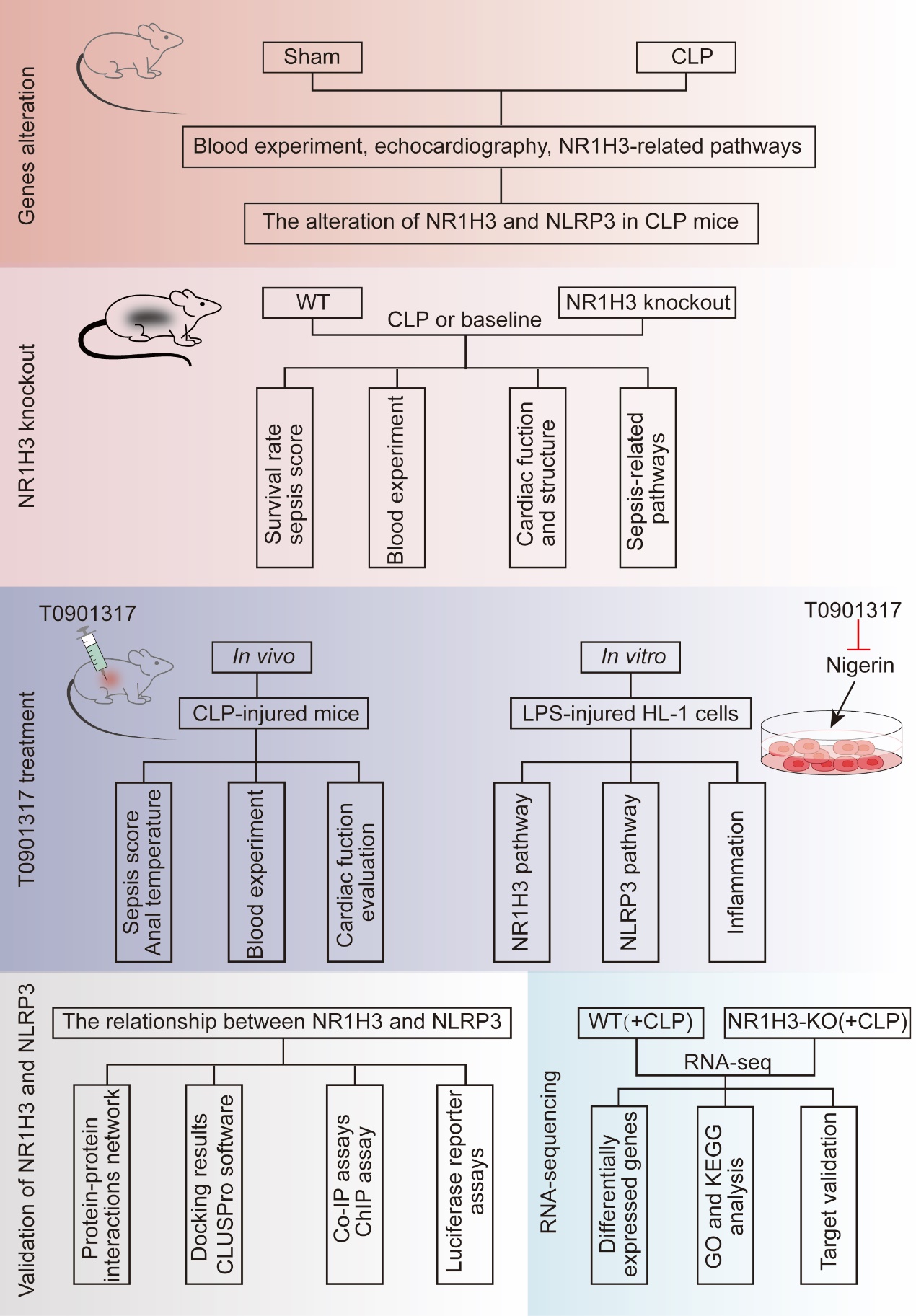


**FIGURE S6** A flowchart to describe the methodology adopted in our study. The workflow includes five parts: 1) The alteration of NR1H3 and NLRP3 in CLP mice was detected; 2) NR1H3 knockout mice with or without CLP were applied to elaborate the role of NR1H3 in septic myocardial injury; 3) T0901317 treatment *in vivo* or *in vitro* alleviated the septic injury, while nigerin exerted negative roles; 4) A series of methods were performed to explore the relationship between NR1H3 and NLRP3; 5) RNA-sequencing identified transcriptome regulated by NR1H3 in septic mice.


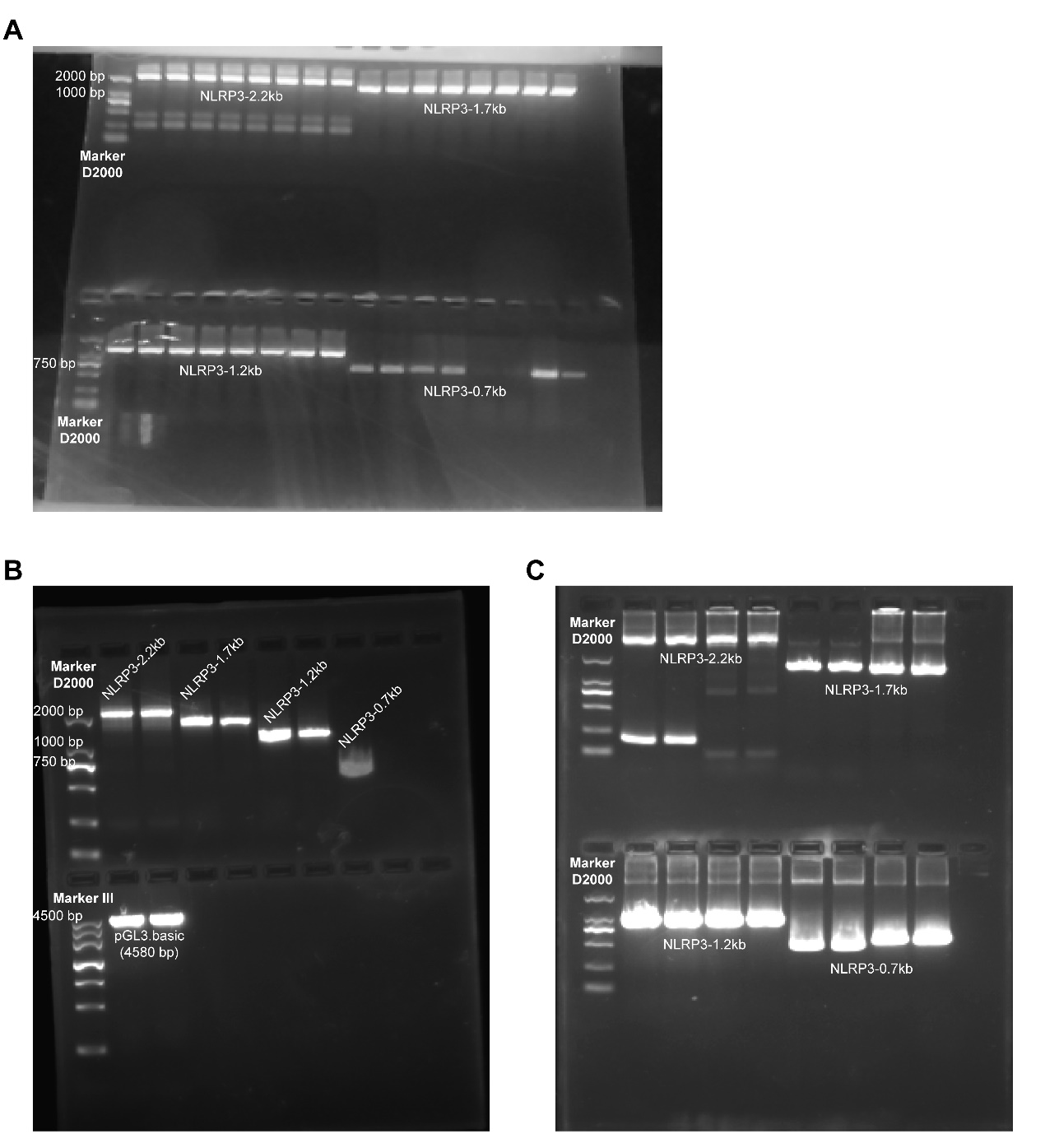


**FIGURE S7** Southern blot in luciferase reporter assays. **(A)** The result of NLRP3 promoters (2.2 kb, 1.7 kb, 1.2 kb, and 0.7 kb) by normal amplified PCR. (**B**) The result of double restriction enzyme digestion by SacI and XhoI. **(C)** The result of successfully constructed pGL3.basic vectors by PCR identification.

**REFERENCE**

1. Shrum B, Anantha RV, Xu SX, et al. A robust scoring system to evaluate sepsis severity in an animal model. BMC Res Notes, 2014. 7(233.

2. Pierce BG, Wiehe K, Hwang H, Kim BH, Vreven T, Weng Z. ZDOCK server: interactive docking prediction of protein-protein complexes and symmetric multimers. Bioinformatics, 2014. 30(12):1771-3.

3. Schlee S, Straub K, Schwab T, Kinateder T, Merkl R, Sterner R. Prediction of quaternary structure by analysis of hot spot residues in protein-protein interfaces: the case of anthranilate phosphoribosyltransferases. Proteins, 2019. 87(10):815-25.
